# Brown adipose tissue dysfunction promotes heart failure via a trimethylamine N-oxide-dependent mechanism

**DOI:** 10.1038/s41598-022-19245-x

**Published:** 2022-09-01

**Authors:** Yohko Yoshida, Ippei Shimizu, Atsuhiro Shimada, Keita Nakahara, Sachiko Yanagisawa, Minoru Kubo, Shinji Fukuda, Chiharu Ishii, Hiromitsu Yamamoto, Takamasa Ishikawa, Kuniyuki Kano, Junken Aoki, Goro Katsuumi, Masayoshi Suda, Kazuyuki Ozaki, Yutaka Yoshida, Shujiro Okuda, Shigeo Ohta, Shiki Okamoto, Yasuhiko Minokoshi, Kanako Oda, Toshikuni Sasaoka, Manabu Abe, Kenji Sakimura, Yoshiaki Kubota, Norihiko Yoshimura, Shingo Kajimura, Maria Zuriaga, Kenneth Walsh, Tomoyoshi Soga, Tohru Minamino

**Affiliations:** 1 Department of Cardiovascular Biology and Medicine, Juntendo University Graduate School of Medicine, Tokyo, 113-8431 Japan; 2 Department of Advanced Senotherapeutics, Juntendo University Graduate School of Medicine, Tokyo, 113-8431 Japan; 3 Department of Applied Life Science, Faculty of Applied Biological Sciences, Gifu University, Gifu, 501-1193 Japan; 4 Graduate School of Science, University of Hyogo, Hyogo, 678-1297 Japan; 5 Institute for Advanced Biosciences, Keio University, 246-2 Mizukami, Kakuganji, Tsuruoka, Yamagata 997-0052 Japan; 6 Intestinal Microbiota Project, Kanagawa Institute of Industrial Science and Technology, Kanagawa, 210-0821 Japan; 7 Transborder Medical Research Center, University of Tsukuba, Ibaraki, 305-8575 Japan; 8 Department of Health Chemistry, Graduate School of Pharmaceutical Sciences, The University of Tokyo, Tokyo, 113-0033 Japan; 9 Department of Cardiovascular Biology and Medicine, Niigata University Graduate School of Medical and Dental Sciences, Niigata, 951-8510 Japan; 10 Department of Structural Pathology, Kidney Research Center, Niigata University Graduate School of Medical and Dental Sciences, Niigata, 951-8510 Japan; 11 Division of Bioinformatics, Niigata University Graduate School of Medical and Dental Sciences, Niigata, 951-8510 Japan; 12 Department of Neurology, Juntendo University Graduate School of Medicine, Tokyo, 113-8421 Japan; 13 Second Department of Internal Medicine (Endocrinology, Diabetes and Metabolism, Hematology, Rheumatology), Graduate School of Medicine, University of the Ryukyus, Okinawa, 903-0215 Japan; 14 Department of Homeostatic Regulation, Division of Endocrinology and Metabolism, National Institutes of Natural Sciences, National Institute for Physiological Sciences, Aichi, 444-8585 Japan; 15 Department of Comparative and Experimental Medicine, Brain Research Institute, Niigata University, Niigata, 951-8585 Japan; 16 Department of Cellular Neurobiology, Brain Research Institute, Niigata University, Niigata, 951-8585 Japan; 17 Department of Animal Model Development, Brain Research Institute, Niigata University, Niigata, 951-8585 Japan; 18 Department of Anatomy, Keio University School of Medicine, Tokyo, 160-8582 Japan; 19 Department of Radiology and Radiation Oncology, Niigata University Graduate School of Medical and Dental Sciences, Niigata, 951-8510 Japan; 20 Department of Radiology, Niigata City General Hospital, Niigata, 950-1197 Japan; 21 Division of Endocrinology, Diabetes & Metabolism, Beth Israel Deaconess Medical Center, Harvard Medical School, Boston, USA; 22 Centro Nacional de Investigaciones Cardiovasculares (CNIC), Madrid, Spain; 23 Division of Cardiovascular Medicine, Robert M. Berne Cardiovascular Research Center, University of Virginia School of Medicine, Charlottesville, VA 22908 USA; 24 Japan Agency for Medical Research and Development-Core Research for Evolutionary Medical Science and Technology (AMED-CREST), Japan Agency for Medical Research and Development, Tokyo, 100-0004 Japan; 25 Department of Cardiovascular Biology and Medicine, Juntendo University Graduate School of Medicine, 2-1-1 Hongo, Bunkyo-Ku, Tokyo, 113-8421 Japan

**Keywords:** Experimental models of disease, Heart failure

## Abstract

Low body temperature predicts a poor outcome in patients with heart failure, but the underlying pathological mechanisms and implications are largely unknown. Brown adipose tissue (BAT) was initially characterised as a thermogenic organ, and recent studies have suggested it plays a crucial role in maintaining systemic metabolic health. While these reports suggest a potential link between BAT and heart failure, the potential role of BAT dysfunction in heart failure has not been investigated. Here, we demonstrate that alteration of BAT function contributes to development of heart failure through disorientation in choline metabolism. Thoracic aortic constriction (TAC) or myocardial infarction (MI) reduced the thermogenic capacity of BAT in mice, leading to significant reduction of body temperature with cold exposure. BAT became hypoxic with TAC or MI, and hypoxic stress induced apoptosis of brown adipocytes. Enhancement of BAT function improved thermogenesis and cardiac function in TAC mice. Conversely, systolic function was impaired in a mouse model of genetic BAT dysfunction, in association with a low survival rate after TAC. Metabolomic analysis showed that reduced BAT thermogenesis was associated with elevation of plasma trimethylamine N-oxide (TMAO) levels. Administration of TMAO to mice led to significant reduction of phosphocreatine and ATP levels in cardiac tissue via suppression of mitochondrial complex IV activity. Genetic or pharmacological inhibition of flavin-containing monooxygenase reduced the plasma TMAO level in mice, and improved cardiac dysfunction in animals with left ventricular pressure overload. In patients with dilated cardiomyopathy, body temperature was low along with elevation of plasma choline and TMAO levels. These results suggest that maintenance of BAT homeostasis and reducing TMAO production could be potential next-generation therapies for heart failure.

## Introduction

The prognosis of severe heart failure (HF) remains unacceptably poor, and there is an urgent need to find a better treatment for this critical condition. Humans are considered to have around 6500 or more metabolites, and evidence indicates that alterations in the level of some metabolites have a close connection with heart failure^1^. Choline and trimethylamine-N-oxide (TMAO) are reported to increase in patients with heart failure and positively correlate with the severity of the New York Heart Association (NYHA) classification^2^. TMAO is also previously reported to promote atherosclerosis^3^. Choline in the diet is metabolized to trimethylamine (TMA) by gut flora and further oxidized into TMAO in liver^3^. Finally, TMAO enhances cholesterol accumulation in atherosclerotic plaque^3^; however, the mechanistic link between TMAO and heart failure remains to be explored.

Brown adipose tissue (BAT) was initially characterized as a thermogenic organ, particularly in small rodents and human infants, but is now well known to be a metabolically active organ with a crucial role in maintaining systemic metabolic health in adult humans^4–6^. It was reported that a high-calorie diet induced impairment of BAT function in a murine model of obesity, leading to systemic glucose intolerance^7^. Systemic metabolic remodeling occurs in patients with heart failure, and low body temperature predicts a poor clinical outcome^8^. In a heart failure with preserved ejection fraction (HFpEF) murine model, BAT function was reported to be reduced^9^. While these reports suggest a potential link between BAT and heart failure, the potential role of BAT dysfunction in HF has not been fully investigated.

Here we show that BAT dysfunction develops with heart failure. This dysfunction led to increased TMAO levels in the circulation and heart. TMAO suppressed mitochondrial complex IV activity and reduced both ATP and phosphocreatine in cardiac tissues. In the advanced stage of heart failure, cardiac tissue becomes unable to utilize metabolites and enters a critical condition described as “out of fuel”^10^. Maintenance of BAT and inhibition of TMAO may be a potential therapy for heart failure.

## Results

### Left ventricular pressure overload reduces thermogenesis of brown adipose tissue

**Figure 1:**
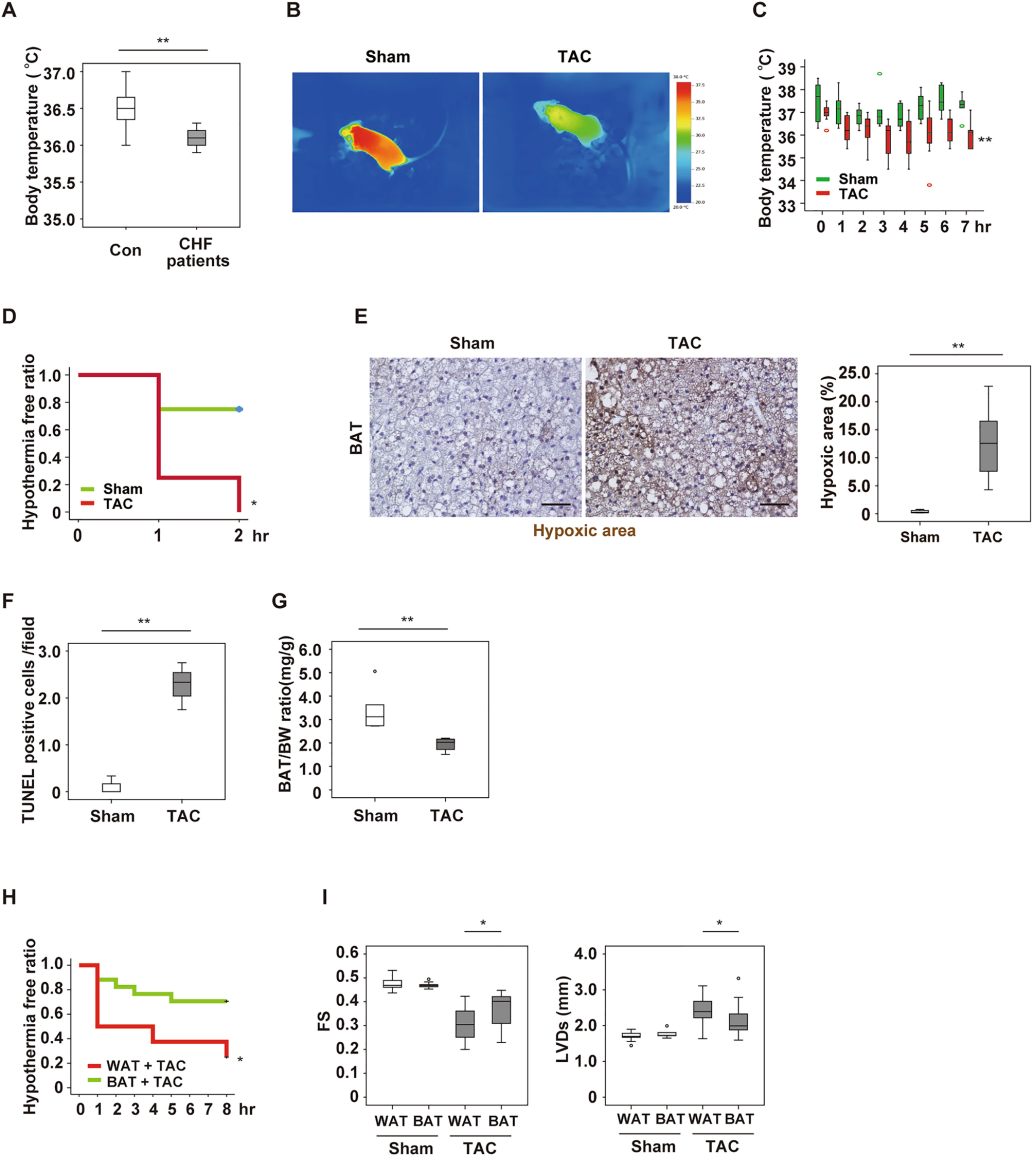
Left ventricular pressure overload induces dysfunction of brown adipose tissue. (**A**) Body temperature of control (Con) subjects and patients with congestive heart failure (CHF) (*n* = 15, 9). (**B**) Surface body temperature measured by a thermal camera in mice at 5 weeks after sham surgery (Sham) or TAC. (**C**) Acute cold tolerance test performed in mice at 4 weeks after Sham or TAC with measurement of body temperature in the scapular region (*n* = 6 and 7, respectively). (**D**) Hypothermia-free ratio during the acute cold tolerance test in mice at 4 weeks after Sham or TAC with measurement of the intraperitoneal temperature (*n* = 4, 4). (**E**) Pimonidazole staining of BAT in mice from (**C**) performed by the Hypoxyprobe-1 method. The right panel shows quantification of the hypoxic area (*n* = 4, 8). Scale bar = 50 μm. (**F**) Quantification of TUNEL-positive cells in BAT from mice at 6 weeks after Sham or TAC (*n* = 3, 3). (**G**) Body weight (BW)-adjusted BAT weight in mice prepared as described in Fig. 1C (*n* = 5, 4). (**H**) Hypothermia-free ratio during the acute cold tolerance test in mice with WAT or BAT transplantation at 2 weeks after TAC (*n* = 11, 17). (**I**) Assessment of cardiac function in mice with WAT or BAT transplantation at 2 weeks after Sham or TAC. FS: fractional shortening (*n* = 10, 11, 9, 19), LVDs: left-ventricular systolic dimension (*n* = 10, 11, 9, 19). Data were analysed by the 2-tailed Student’s t-test (**A**, **E**, **F**, **G** and **I**), repeated measures followed by Tukey’s multiple comparison test (**C**), or the log-rank test for Kaplan–Meier method (**D**, **H**). *P < 0.05, **P < 0.01. Values are shown as the mean ± s.e.m.

In agreement with the previous report^8^, we found that hospitalized patients with heart failure had a lower body temperature than a control group (Fig. 1A, Supplementary Fig. 1A,B). Because BAT has a critical role in maintaining body temperature^4–6^, we investigated how cardiac dysfunction could impact on BAT in two murine models of heart failure. In the first model, thoracic aortic constriction (TAC) was performed in wild-type (WT) mice at 11 weeks of age to generate left ventricular (LV) pressure overload as reported previously^11^. LV dysfunction developed 4 weeks after TAC (Supplementary Fig. 1C–E) in association with significant reduction of both the body surface and intraperitoneal thermogenic responses (Fig. 1B–D, Supplementary Fig. 1F). Body weight and food intake were comparable between the sham and TAC groups, but BAT became hypoxic with an increase of apoptotic cells, and BAT weight was significantly reduced after development of LV pressure overload (Fig. 1E–G and Supplementary Fig. 1G–K). Uncoupling protein-1 (UCP-1) is a proton channel in the inner mitochondrial membrane and uncouples the electron transport chain to generate heat instead of adenosine triphosphate (ATP) in BAT^12^. Cold exposure was reported to introduce inducible brown adipocyte like phenotype in white adipose tissue (WAT)^13^, and nowadays brown adipocyte like cells in white adipose tissues are described as beige cells^14^. We found that UCP-1 in BAT reduced with heart failure (Supplementary Fig. 2A), and level of beige markers remained low in subcutaneous white adipose tissue (Supplementary Fig. 2B). To further characterize BAT in heart failure, we generated another murine model with heart failure. In the second heart failure model, cardiac dysfunction was induced by myocardial infarction (MI) at 11 weeks of age, also resulting in reduced BAT thermogenesis together with accumulation of apoptotic cells in this tissue 6 weeks after MI (Supplementary Fig. 2C–K). The results obtained in these two models suggested a close relation of heart failure to BAT dysfunction and impaired thermogenesis.

Next, we sought to generate a murine model with enhanced BAT function by the transplantation of donor-derived BAT into intraperitoneal cavity of recipient mice. For this aim, WT mice were used as donor or recipient mice. BAT or WAT was transplanted into a visceral cavity of WT mice at 9 weeks of age, and TAC was performed at 11 weeks of age. At 2 weeks after TAC, mice were subjected to analyses. Transplantation of BAT improved cardiac dysfunction and the thermogenic response compared with WAT implantation model (Fig. 1H,I and Supplementary Fig. 2L–O).

### Brown adipose tissue dysfunction deteriorates cardiac function after TAC

**Figure 2:**
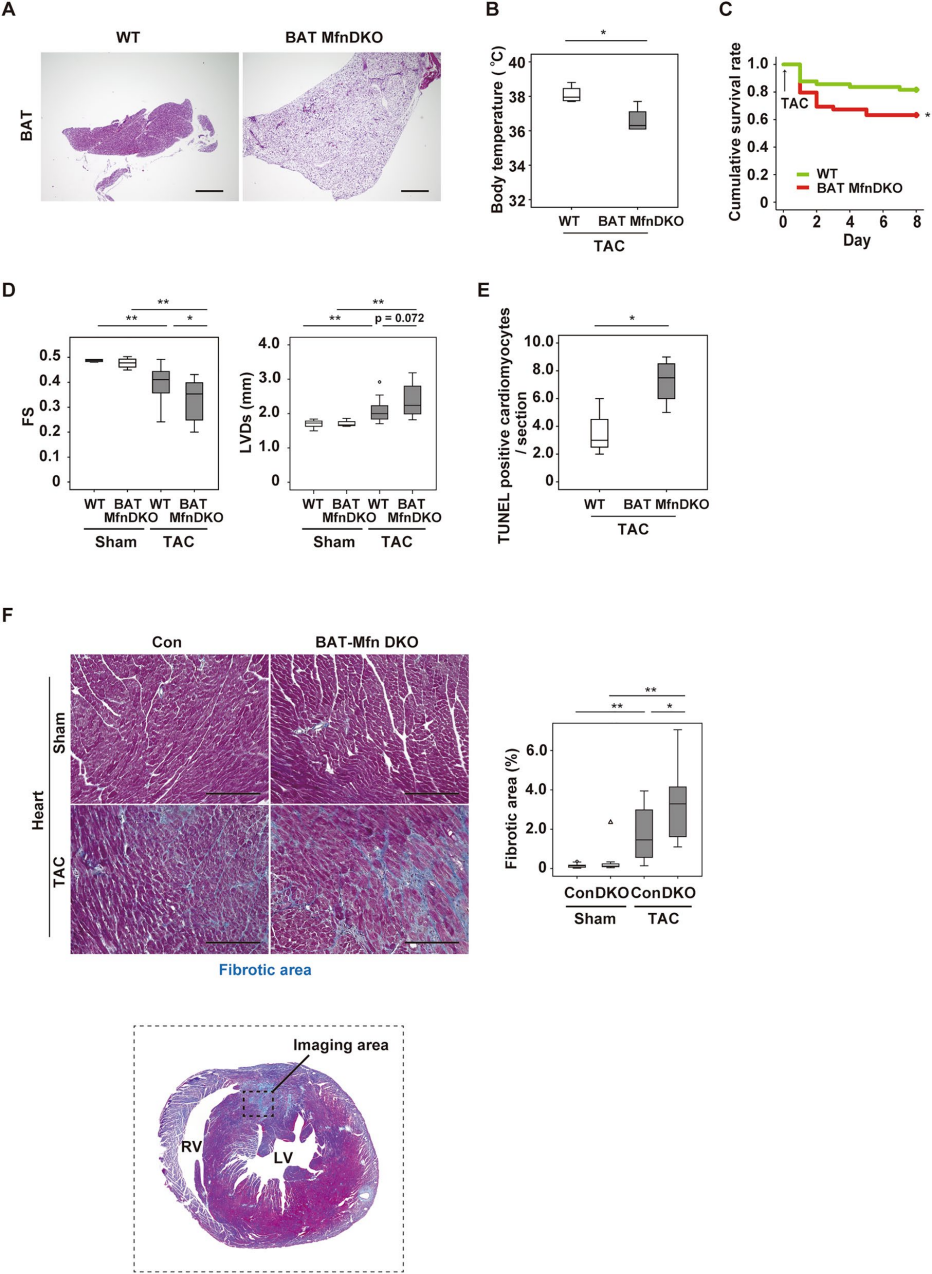
Brown adipose tissue dysfunction deteriorates cardiac function after TAC. (**A**) Hematoxylin and eosin (HE) staining of BAT from 8-week-old littermate control (WT) mice or BAT-specific Mfn1/Mfn2 DKO (BAT Mfn DKO) mice. Scale bar = 500 μm. (**B**) Body temperature of mice at 2 weeks after TAC (*n* = 4, 4). (**C**) Cumulative survival rate of mice at 1 week after TAC (*n* = 49, 49). (**D**) Assessment of cardiac function in the indicated mice at 1 week after Sham or TAC (FS; *n* = 5, 4, 28, 16, LVDs; *n* = 5, 4, 28, 16). (**E**) Quantification of TUNEL-positive cardiomyocytes in the hearts of mice at 1 week after TAC (*n* = 4, 4). (**F**) Masson’s trichrome staining of hearts from the mice. The right panel shows quantification of the fibrotic area (*n* = 13, 10, 21, 13). Scale bar = 50 μm. Data were analysed by the 2-tailed Student’s t-test (**B**, **E**), 2-way ANOVA followed by Dunnett’s comparison test (**D**), or the log-rank test for Kaplan–Meier method (**C**). *P < 0.05, **P < 0.01. Values are shown as the mean ± s.e.m.

To further assess the role of altered BAT function in heart failure, we generated a BAT specific loss-of-function model. For this aim, we crossed Ucp1-Cre mice with *Mfn1*^flox/flox^ and *Mfn2*^flox/flox^ mice to obtain BAT Mfn double knockout (DKO) mice. Mitofusin1 and 2 (encoded by *Mfn1* and *Mfn2* respectively) are critically involved in the mitochondrial fusion process, and depletion of both *Mfn1* and *Mfn2* induced whitening of BAT (Fig. 2A and Supplementary Fig. 3A–C). TAC was performed in BAT Mfn DKO mice and their littermate control mice at 11 weeks of age, and mice were subjected to analyses at 1 week after TAC. After LV pressure overload was induced, BAT Mfn DKO mice showed a lower body temperature, higher mortality, and worse cardiac function than littermate control mice (Fig. 2B–D). Apoptotic cardiomyocytes and cardiac fibrosis were also increased in BAT Mfn DKO mice (Fig. 2E,F). However, BAT Mfn DKO and littermate control mice showed comparable body weight, food intake, and cardiac function under basal conditions (Supplementary Fig. 3D–F). In addition, we generated another BAT loss-of-function model by surgical removal of interscapular BAT (BATectomy). BAT was removed at 10 weeks of age, and TAC was performed at 11 weeks of age. At 2 weeks after TAC, mice were subjected to analyses. We confirmed the reproducible results in the BATectomy model (Supplementary Fig. 3G–L). Chronic exposure to cold (15 °C) for 1 week at 2 weeks after TAC had no effect on the cardiac changes after TAC in BAT *Mfn*DKO mice or WT mice, suggesting that modulation of environmental temperatures had minor effects on HF (Supplementary Fig. 4A–F).

It is well accepted that sustained activation of the sympathetic nervous system promotes cardiac dysfunction^15,16^. Because we found an increase of norepinephrine in BAT after TAC (Supplementary Fig. 4G), we examined the effects of chronic adrenergic stimulation on BAT function. We found that two-weeks infusion of isoproterenol significantly impaired thermogenic response to acute cold exposure (Supplementary Fig. 4H) without inducing cardiac dysfunction (Supplementary Fig. 4I), suggesting that chronic activation of adrenergic signaling during LV pressure overload induces BAT dysfunction which promoted cardiac dysfunction.

### Choline and trimethylamine N-oxide are increased with heart failure

**Figure 3:**
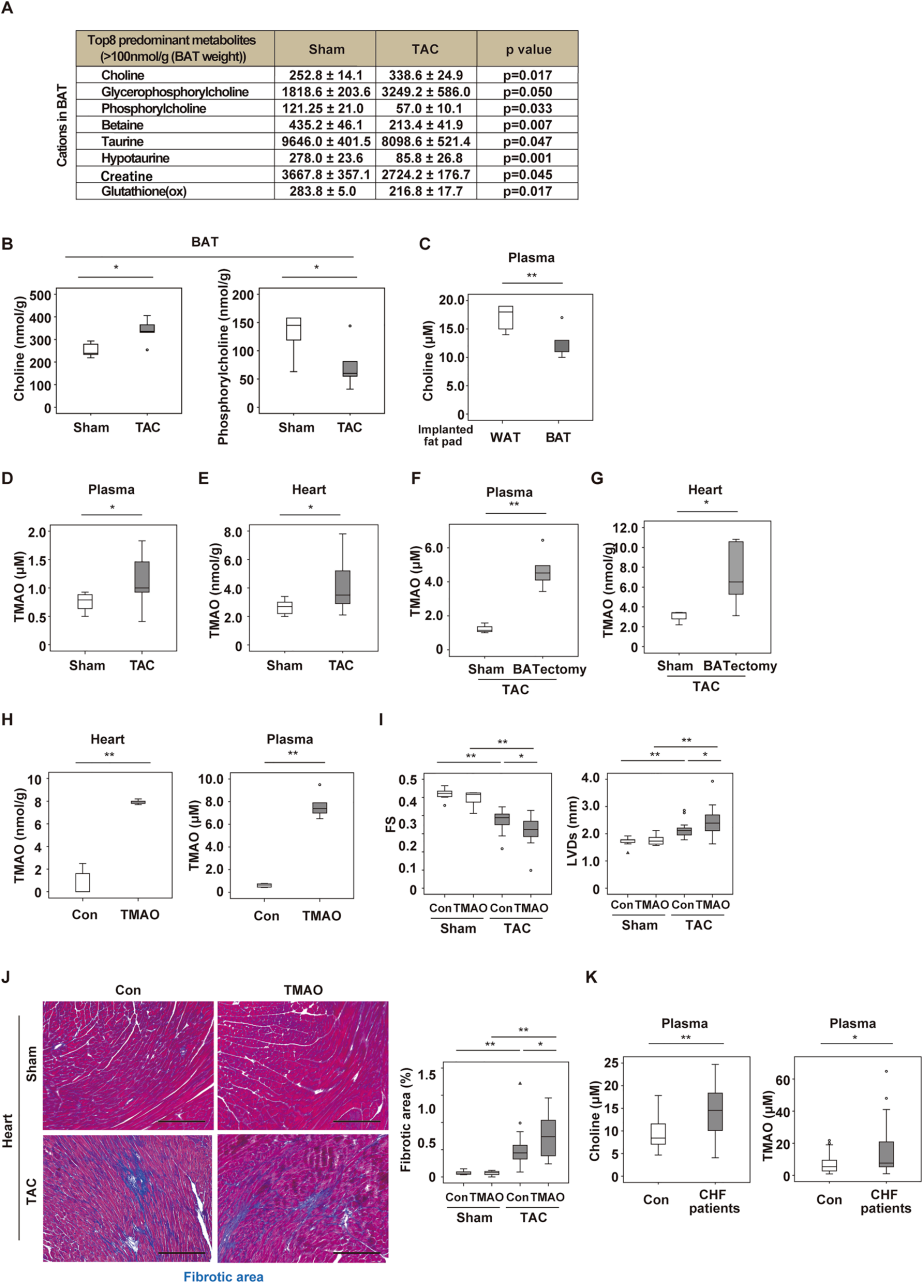
Choline and trimethylamine N-oxide are increased in heart failure. (**A**) Changes of cationic metabolites in BAT from mice at 2 weeks after TAC or sham surgery (Sham) assessed by CE-TOF/MS. Results of some metabolites are also shown in Fig. 3B as a box plot panel. (**B**) Tissue weight-adjusted levels of choline (*n* = 5, 5) or phosphorylcholine (*n* = 5, 5) in BAT from mice at 2 weeks after Sham or TAC. (**C**) Plasma choline level in mice with implantation of WAT or BAT (*n* = 5, 5). (**D**, **E**) Plasma (**D**) or myocardial (**E**) trimethylamine N-oxide (TMAO) level in mice at 2 weeks after sham or TAC (*n* = 11, 11). (**F**, **G**) Plasma (**F**) or heart (**G**) TMAO level in sham and BATectomy model mice 2 weeks after TAC (*n* = 3, 5). (H) TMAO levels in myocardium (left) and plasma (right) of WT mice treated with PBS (Con) or TMAO mice for 2 weeks (*n* = 5, 5). (**I**) Cardiac function of mice at 2 weeks after Sham or TAC with/without TMAO treatment (*n* = 7, 7, 19, 16). (**J**) Masson’s trichrome staining of hearts from mice in (**I**). The right panel shows quantification of the fibrotic area (*n* = 7, 7, 21, 17). Scale bar = 50 μm. (**K**) Plasma choline level (left panel) and TMAO level (right panel) in control subjects (Con) or patients with CHF (*n* = 23, 30). Data were analysed by the 2-tailed Student’s t-test (**A**–**H** and **K**) or 2-way ANOVA followed by Dunnett’s comparison test (**I** and **J**). *P < 0.05, **P < 0.01. Values are shown as the mean ± s.e.m.

To clarify the mechanism by which alteration in BAT function promotes heart failure, we analyzed the levels of various metabolites in our models using CE-TOF/MS. Among cationic metabolites, we found an abundance of choline and choline-related metabolites in BAT (Fig. 3A) at 2 weeks after TAC (13 weeks of age). Notably, choline was increased in BAT after TAC, while phosphorylcholine was decreased (Fig. 3B). Metabolomic flux studies analyzing d9-choline showed that choline was incorporated by differentiated brown adipocytes and was metabolized further in the in vitro setting (Supplementary Fig. 5A,B). At 4 weeks after transplantation of BAT or WAT, BAT transplantation without TAC led to a significant decrease of the plasma choline level (Fig. 3C). Recent studies have demonstrated that choline and its metabolites (including TMAO) are increased in patients with heart failure, showing a positive correlation with the severity of heart failure^2^. Consistent with previous reports, we found an increase of both plasma and cardiac TMAO levels 2 weeks after TAC (Fig. 3D,E). We also tested this in BATectomy model. BAT was removed at 10 weeks of age, and TAC was performed at 11 weeks of age. At 2 weeks after TAC, we analyzed plasma and heart samples and found that an increase of TMAO was enhanced in BATectomy with TAC than control with TAC (Fig. 3F,G). These data suggested that circulating choline is taken up and metabolized by healthy BAT under physiological conditions, possibly to maintain the cell membrane integrity of brown adipocytes, while unprocessed choline is oxidized to TMAO after BAT dysfunction occurs.

Next, we determined the direct effect of TMAO on the failing heart. We continuously infused TMAO into WT mice for 2 weeks since 11 weeks of age and found that continuous infusion of TMAO led to a significant increase of the heart and plasma TMAO level (Fig. 3H). We also generated TAC model mice with TMAO infusion. We performed TAC operation in WT mice at 11 weeks of age. Two weeks after TAC, we started continuous infusion of TMAO and analyzed them additional 2 weeks later (totally 4 weeks after TAC). We found that TMAO treatment exacerbated cardiac dysfunction and fibrosis during pressure overload (Fig. 3I,J). In addition, plasma choline and TMAO levels were higher in patients with heart failure compared to controls (Fig. 3K).

It is reported that dietary choline is metabolized to trimethylamine (TMA) by gut microbiota, after which TMA is further metabolized to TMAO by flavin-containing monooxygenase (FMO)^17^. Therefore, we generated high choline-fed TAC mice model. At 2 weeks after TAC, WT mice were subjected to administration of a high- (1%) or low-choline (0%) diet for 2 weeks. Maintaining WT mice on a high-choline diet increased TMAO levels in plasma and myocardium, reduced cardiac function, and increased cardiac fibrosis (Supplementary Fig. 6A–G). These results suggest the contribution of increased TMAO during LV pressure overload in the progression of heart failure.

### Inhibition of FMO ameliorates cardiac dysfunction during left ventricular pressure overload

**Figure 4:**
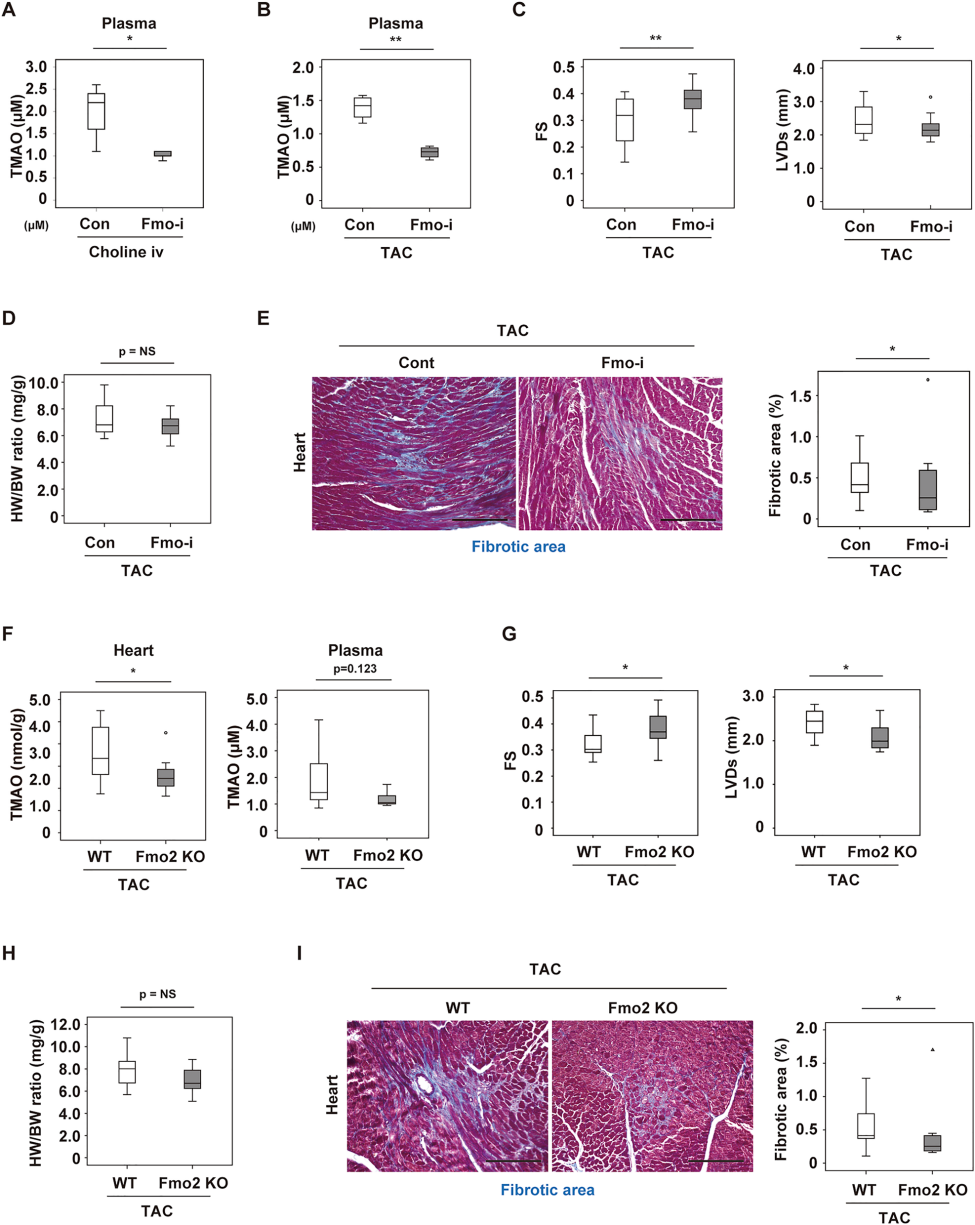
Inhibition of FMO ameliorates cardiac dysfunction during left ventricular pressure overload. (**A**) Plasma TMAO level in WT mice after intravenous choline infusion (500 nmol, 6 h), without (Con) or with the administration of 0.05% methimazole (Fmo-i) (*n* = 5, 5). Fmo-i was administered through drinking water for 1 week, and then choline infusion was performed. (**B**) Plasma TMAO level in mice at 3 weeks after TAC (*n* = 4, 4). For this study, 0.05% methimazole (Fmo-i) was administered in drinking water 1 week after TAC operation for 2 weeks total. Experiments were performed 3 weeks after TAC. (**C**) Cardiac function of mice (FS: fractional shortening, LVDs: left ventricular systolic dimension; *n* = 22, 21). (**D**) Body weight-adjusted heart weight of mice (n = 22, 21). (**E**) Masson’s trichrome staining of hearts from mice. The right panel shows quantification of the fibrotic area (n = 11, 14). Scale bar = 50 μm. (**F**) TMAO levels in hearts or plasma from littermate wild-type (WT) or systemic *Fmo2* knockout (*Fmo2* KO) mice at 2 weeks after TAC (*n* = 8, 9). (**G**) Cardiac function of mice prepared as described in Fig. 4F (fractional shortening (FS); *n* = 14, 13, left ventricular systolic dimension (LVDs); *n* = 14, 13). (**H**) Body weight-adjusted heart weight of mice (*n* = 14, 13). (**I**) Masson’s trichrome staining of hearts. The right panel shows quantification of the fibrotic area (*n* = 12, 8). Scale bar = 50 μm. Data were analysed by the 2-tailed Student’s t-test (A-I). *P < 0.05, **P < 0.01. Values are shown as the mean ± s.e.m. NS = not significant.

We next examined the effects of FMO inhibition on heart failure using methimazole, a pan FMO inhibitor (Fmo-i)^18–20^. Fmo-i was administered WT mice since 11 weeks of age through drinking water for 1 week, and then choline infusion was performed. We found FMO inhibition effectively reduced circulating TMAO levels in the intravenous choline injection model (Fig. 4A). This treatment also decreased the plasma TMAO level after TAC together with improvement of cardiac dysfunction and fibrosis (Fig. 4B–E).

There are five isoforms of flavin-containing monooxygenase in mammals, which are designated as Fmo1-5, and it has been reported that their expression varies among different cells and tissues as well as with gender and developmental stage^21,22^. The previous studies demonstrated that Fmo3 played a critical role in female mice, but male mice were known to have low Fmo3 expression^22^. Using bioinformatic analysis, we identified Fmo2 as a candidate enzyme for TMAO production in male mice. Thus, we generated a genetic model of systemic Fmo2 depletion in mice (Fmo2 KO mice), and performed TAC operation in these mice at 11 weeks of age. We found reduction of the cardiac TMAO level in these mice 2 weeks after TAC, along with improvement of cardiac function and fibrosis despite no change of heart weight (Fig. 4F–I). These findings suggested that Fmo2 has a causal role for TMAO production in the presence of cardiac pressure overload and that inhibition of Fmo2 would be therapeutic target for heart failure.

### Trimethylamine N-oxide inhibits mitochondrial respiration in the heart

**Figure 5:**
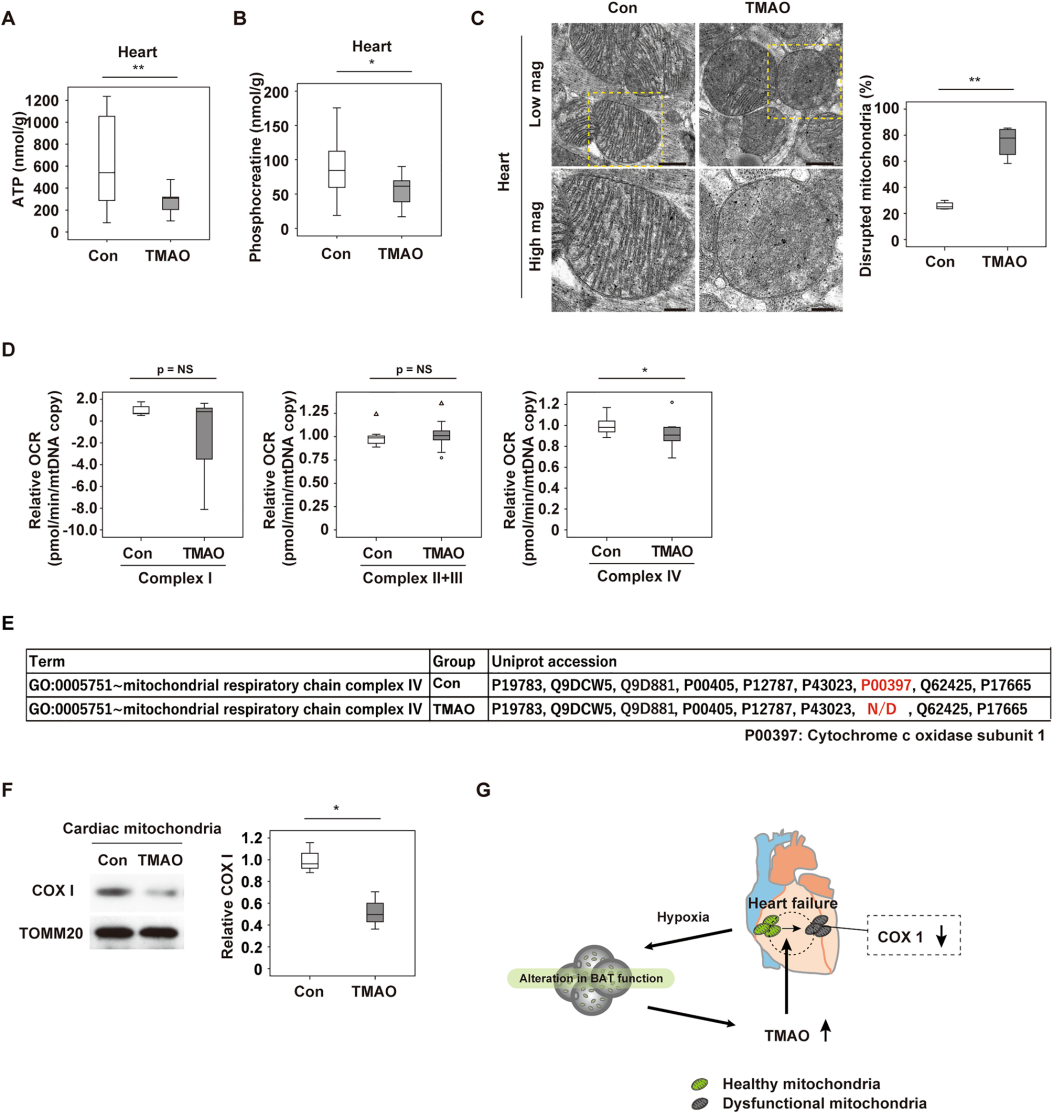
Trimethylamine N-oxide inhibits mitochondrial respiration in the heart. (**A**, **B**) Metabolomic study analysing ATP (**A**) (*n* = 24, 11) or phosphocreatine (**B**) (*n* = 24, 11) in the hearts of WT mice administered PBS (Con) or Trimethylamine N-oxide (TMAO) for 2 weeks. (**C**) Transmission electron microscopy of cardiac tissues from mice prepared as described in Fig. 5A. The right panel shows quantification of the disrupted mitochondria (*n* = 4, 4). Scale bar = 500 nm for low magnification and 200 nm for high magnification. (**D**) Oxygen consumption rate (OCR) assessing respiration by the indicated complexes in mitochondria isolated from the cardiac tissues of mice prepared as described in Fig. 5A (*n* = 7, 9). (**E**) Mitochondrial complex IV proteins of cardiac mitochondria assessed by mass spectrometry in the PBS (Con) or TMAO treated WT mice. N/D = Not detected. (**F**) Western blot analysis of COX1 in cardiac mitochondria prepared as described in (**E**). TOMM20 was used as the loading control. The right panel shows quantification of the data (*n* = 3, 3). Original blots are presented in Supplementary Fig. 9. (**G**) Graphical abstract showing a summary of the findings. Data were analyzed by the 2-tailed Student’s t-test (**A**, **B**, **C**, **D** and **F**). *P < 0.05, **P < 0.01. Values represent the mean ± s.e.m. *NS* not significant.

Next, we sought to elucidate the underlying mechanism of TMAO-induced cardiac dysfunction. We infused TMAO into 11 weeks old WT mice for 2 weeks. Administration of TMAO to WT mice significantly reduced cardiac tissue levels of ATP and phosphocreatine (Fig. 5A,B, Supplementary Fig. 7A). Electron microscopy of these mice revealed disruption of mitochondrial cristae in hearts obtained from mice with TMAO infusion and this was enhanced with LV pressure overload (Fig. 5C, Supplementary Fig. 7B). TAC led to similar pathological changes of cardiac mitochondria, and this mitochondrial disruption was augmented in BAT *Mfn* DKO and BATectomy models, and showed synergistic mitochondrial morphological alteration with TMAO administration in WT mice (Supplementary Fig. 7B–D). We also demonstrated that disarray of mitochondrial cristae in response to LV pressure overload was reduced in Fmo2 KO mice (Supplementary Fig. 7E). Extracellular flux analysis of cardiac mitochondria isolated from WT mice with TMAO infusion showed reduction of oxidative phosphorylation and mitochondrial complex IV function (Fig. 5D).

To further investigate the underlying mechanism, we conducted RNA-sequencing (seq) analysis of cardiac tissues and proteomic analysis of cardiac mitochondria. We infused TMAO into WT mice at 11 weeks of age for 2 weeks, and then isolated cardiac mitochondria from these mice for RNA-seq or proteomics analyses. Our RNA-seq analysis indicated the occurrence of metabolic remodeling in cardiac tissues (Supplementary Fig. 8A), while the proteomic study identified cytochrome c oxidase subunit 1 (COXI) protein (also known as MT-CO1 and encoded by *Mtco1*) in cardiac mitochondria from control mice, but not in mice with TMAO infusion (Fig. 5E, Supplementary Fig. 8B). Western blot analysis and extracted ion chromatograms of two peptides unique to COXI protein revealed approximately 50% reduction of this protein by TMAO infusion (Fig. 5F). Quantitative PCR showed that *Mtco1*, *Mtco2* and *Mtco3* transcripts were not reduced by TMAO administration (Supplementary Fig. 8C), suggesting that TMAO inhibited mitochondrial respiration in the heart through the post-transcriptional regulation of mitochondrial complex IV protein.

## Discussion

In the present study, we demonstrated that BAT dysfunction develops in murine models of heart failure and leads to abnormal choline metabolism. In turn, BAT dysfunction promotes heart failure through the production of the choline metabolite TMAO. Although Fmo3 was reported to have a critical role for TMAO production in female mice, it is well known that sex differences exist in the expression profiles of FMOs^22^. In this study, we adopted a bioinformatic approach and focused on Fmo2. Studies performed in male mice with genetic inhibition of Fmo2 showed that this enzyme has a critical role in converting choline to TMAO during heart failure, and revealed that TMAO suppresses cardiac metabolism by inhibiting mitochondrial complex IV. It is generally accepted that the intestinal flora converts dietary choline to TMA, after which TMA is metabolized to TMAO in the liver^17^. However, it remains to be determined how BAT dysfunction is linked to increased levels of TMAO and whether heart failure affects the activity of any FMOs.

Accumulating evidence indicates bacterial dysbiosis develops in patients with heart failure^23^. Pasini et al. reported that heart failure leads to an increase in pathogenic bacterial colonies including Candida, Campylobacter, Shigella, Salmonella, Yersinia enterocolitica^24^. Heart failure patients were also shown to have a decrease in *Faecalibacterium prausnitzii* and an increase in *Ruminococcus gnavus* and were associated with an increase in TMA-lyase, a key enzyme for TMAO generation^25^. Hayashi et al. showed positive correlations between the abundance of the genus Escherichia/Shigella and the level of TMAO^26^. Analyzing dysbiosis in murine heart failure models, testing the level of TMA-lyase, and showing the potential link among FMOs would be an interesting research topic to be explored.

We found FMO2 inhibition ameliorated cardiac dysfunction in mice. FMOs are known as regulators of stress resistance and play a role in xenobiotic-detoxifying and drug metabolism^27^. Endogenous function of FMOs are less clear, and it remains open question to be tested whether FMOs become a therapeutic target^27^. In the present study, we did not analyze the activity of BAT in patients with heart failure. Tahara et al. reported the results of [^18^F]-fluorodeoxyglucose-positron emission tomography in a 23-year old female patient with heart failure who had low body temperature and suggested insufficient BAT-induced thermogenesis in this patient^28^. In our left ventricular pressure overload model with reduced ejection fraction, we found the diminished skin temperature at interscapular area where BAT predominantly exists. Thermogenic response under acute cold tolerance was also reduced. Under this condition, we found BAT becomes hypoxic together with an increase in apoptotic cells in this organ. Adrenergic activation is well known to increase with heart failure^15,16^. In our TAC model, norepinephrine (NE) increased in BAT, and we found catecholamine infusion resulted in diminished thermogenesis. In this model, left ventricular hypertrophy developed, but systolic function was preserved. These data suggest that continuous activation of catecholamine signaling initially promotes BAT dysfunction. This would increase TMAO level, enhancing cardiac dysfunction, leading to a vicious cycle of BAT and cardiac dysfunction. Since an increase in TMAO levels can potentially cause dysfunction of multiple organs with abundant mitochondria, this metabolite may play a role in sarcopenia and other systemic effects of heart failure. Thus, additional research on the role of BAT and abnormal choline metabolism in heart failure may lead to a better understanding of this complex condition (Fig. 5G).

This study has several limitations. We have not provided clinical evidence showing that BAT dysfunction is associated with heart failure. BAT Mfn DKO mice were on a mixed background. Methimazole is not a specific inhibitor for FMOs. Only male mice were utilized for all experiments.

## Methods

### Human samples

Blood samples and clinical data were collected from patients of Niigata University Hospital. All subjects provided written informed consent prior to participation in these studies. The Scientific‐Ethics Committees of Niigata University approved the protocols of all the studies (protocol number 2015-2292, 2017-0102), and each investigation was performed in accordance with the Declaration of Helsinki. Blood samples were immediately centrifuged to obtain plasma, which was subjected to CE-TOF/MS at Keio University. For body temperature studies, data were collected from admitted male Asian patients who were registered to our biobank between year 2012–2015. Analyses for the control group were done for patients who had EF > 50% and diagnosed as either (n = 3), arrhythmias (n = 10) or hypertension (n = 2). Analyses for the heart failure group were done for patients who were diagnosed as (n = 8) or dilated phase hypertrophic cardiomyopathy (n = 1). For metabolome studies analyzing TMAO or choline in plasma of control or heart failure patients, data for these were collected from metabolomic biobank generated with samples collected between year 2015–2018 in Niigata University. The control group included vasospastic angina (n = 10), paroxysmal supraventricular tachycardia (n = 6) and other cases including syncope with unknown reasons (n = 7). The heart failure group included idiopathic dilated cardiomyopathy (n = 6), dilated phase hypertrophic cardiomyopathy (n = 5), ischemic cardiomyopathy (n = 17) and other cases including amyloidosis (n = 2).

### Animal models

All animal experiments were conducted in compliance with the guidelines which was reviewed by the Institutional Animal Care and Use Committee of Niigata University and Juntendo University, and this study is approved by the Institutional Animal Care and Use Committee of Niigata University and Juntendo University. The study was carried out in compliance with the ARRIVE guidelines. Mice were housed in the animal facilities at Niigata University under specific pathogen-free conditions at a constant temperature of 23 °C, and a 12 h light/12 h dark cycle. Investigators were blinded to mouse genotypes during experiments. All mice were randomly allocated for surgical procedures under blinded condition. Before surgical procedures, mice are anesthetized with a cocktail of medetomidine hydrochloride (0.75 mg/kg, i.p.), midazolam (4 mg/kg, i.p.) and butorphanol tartrate (5 mg/kg, i.p.). TAC and MI were performed in 11-week-old male mice, as described previously^11^, and evaluation was done 4 weeks after surgery unless mentioned otherwise. Sham-operated mice underwent the same procedures, except for aortic constriction or coronary artery ligation. Transplantation of BAT or WAT was done as previously reported^29^. Surgical removal of BAT (BATectomy) was done as previously reported^30^. In some experiments, mice were housed at 15 °C (cold exposure) for 1 week with free access to water and chow under a 12 h light/12 h dark cycle. In some experiments, WT mice were fed a high-choline diet (10 g/kg, Dyets Inc., Bethlehem PA, USA) or low-choline diet (0 g/kg, Dyets Inc.). Wild-type mice were on a C57BL/6NCrSlc background. Mice expressing Cre recombinase in Ucp1-positive cells (Ucp1-Cre)(C57BL/6 J background)^31^ were crossed with mice carrying floxed *Mfn1* alleles and mice carrying floxed *Mfn2* alleles with a 129/ Black-Swiss/C57BL/6 background^32^ to generate mice with brown adipose tissue (BAT)-specific double knockout of Mfn1 and Mfn2 (BAT-Mfn1/2 DKO mice). The genotype of the littermate controls was Ucp1-Cre^–^: *Mfn1*^flox/flox^: *Mfn2*^flox/flox^. We used the CRISPR/Cas9 technique targeting exon 3 of the *Fmo2* allele to generate Fmo2-deficient mice (with a C57BL/6NCrSlc background). In some experiments, 500 nmol of choline (Sigma, 292257) was injected via the tail vein and samples were collected after 6 h. In some experiments, isoproterenol (Sigma, I-6504; 30 mg/kg/day) or trimethylamine N-oxide (TMAO: Sigma, 317594; 25 mg/kg/day) was administered via an infusion pump (Alzet). Methimazole (Sigma, M-8506), pan FMO inhibitor (Fmo-i), was administered in the drinking water at a concentration of 0.05%.

### Cell culture

The brown pre-adipocyte cell line was a kind gift from Dr. C. Ronald Kahn (Joslin Diabetes Center and Harvard Medical School, Section on Integrative Physiology and Metabolism, Boston, USA)^31,33^. This cell line was established from wild-type FVB mice and was immortalized by infection with the pBabe retroviral vector encoding SV40T antigen. Cells were cultured in high glucose DMEM (Gibco, 12430) with 10% fetal bovine serum (FBS) and 100 U/ml penicillin/streptomycin solution (P/S), and differentiation was induced as described previously^34^. Fully differentiated brown adipocytes were used for analysis after 10 days of culture.

### Acute cold exposure

Body temperature was assessed by subcutaneous implantation of a biocompatible sterile microchip transponder (IPTT-300 Extended Accuracy Calibration; Bio Medic Data Systems) in the scapular region or intraperitoneally according to the manufacturer’s instructions. The cold tolerance test (CTT) was performed at 4 °C and body temperature of the animals was measured at hourly intervals for 8 h. Hypothermia was defined as a temperature < 35 °C in Supplementary Fig. 3I, < 32 °C in Fig. 1D or < 30 °C in Figs. 1H and 2C. In some experiments, a tail tip cold exposure (TT-CE) test was performed. Before the TT-CE study, pelage hair on the backs of the mice was removed with a depilatory. The distal tails (approximately the distal 5 mm) were exposed to ice water for 10 min, and interscapular skin temperature was assessed with an infrared thermography camera (Testo, Inc., Sparta NJ, USA, 885) according to the manufacturer’s instructions.

### Physiological analyses

Mice were housed individually and their body weight and food intake were monitored. Surface body temperature was measured using a thermal camera (Testo, 885) as indicated by the manufacturer. Echocardiography was performed with a Vevo 2100 High Resolution Imaging System (Visual Sonics Inc., Toronto, Ontario, Canada).

### Metabolomic analyses

Metabolomic analyses were done by Soga et al. using capillary electrophoresis time-of-flight/mass spectrometry (CE-TOF/MS), as described previously^35^. Lipidomic flux analysis was performed with deuterated choline (choline chloride-(trimethyl-d9); Taiyo Nippon Sanso, 492051). Fully differentiated brown adipocytes were cultured with deuterated choline. Lipidomic analyses were done by Ishikawa et al. using liquid chromatograph time-of-flight/mass spectrometry (LC-TOF/MS), as described previously^36,37^. For in vivo metabolomic analysis, BAT and hearts were excised from the mice and immediately frozen in liquid nitrogen. Mouse and human blood samples were immediately centrifuged, and 40 μl of plasma was mixed with 360 μl of methanol containing L-methionine sulfone, MES, and CSA (all at 20 μM). Then the aqueous layer was extracted with chloroform and filtered, before being subjected to CE-TOF/MS.

### Histological and physiological analyses

Cardiac tissue and brown adipose tissue samples were harvested, fixed overnight in 10% formalin, embedded in paraffin, and sectioned for immunohistochemistry or hematoxylin–eosin (HE) staining. Fibrosis was detected with Masson’s trichrome stain and four fields per section were randomly selected for quantification with the ImageJ system. Tissue hypoxia was estimated with the Hypoxyprobe-1 (Hypoxyprobe Inc., HPI-100) according to the manufacturer’s instructions. Briefly, pimonidazole (60 mg/kg) was injected intraperitoneally at 90 min before sacrifice, after which BAT was harvested and fixed in 10% formalin overnight. Then BAT was embedded in paraffin, sectioned, and stained with the Hypoxyprobe-1 monoclonal antibody (which binds to protein adducts of pimonidazole in hypoxic cells). The sections were counterstained with hematoxylin. TUNEL labeling was performed according to the manufacturer’s protocol (In Situ Cell Death Detection Kit, Fluorescein; Roche, 1684795) in combination with WGA-lectin (Sigma, L5266) and Hoechst (Life Technologies, 33258). The tissue sections were stained with anti-Hif1α antibody (Abcam, ab179483), Triticum vulgaris (wheat) lectin-Alexa Fluor 488 conjugate for staining cell membranes (Thermo Fisher, W11261), and Hoechst (Life Technologies, 33258). The secondary antibody for anti-Hif1α antibody was goat anti-rabbit IgG H&L (Cy5) (Abcam, ab97077). All primary and secondary antibodies were used at a dilution of 1:50, except for Hoechst (1:1000). Stained sections were photographed with a Biorevo (Keyence Co.) or a confocal microscopy (C2+, Nicon Co.). For electron microscopy, heart tissue was fixed in 2.5% glutaraldehyde. Grids for electron microscopy were prepared by Masaaki Nameta at the core electron microscope facility of Niigata University, and electron microscopy was done at Niigata University Medical Campus using a JEM1400 TEM. The disrupted mitochondria were defined as ones structurally destroyed or whose cristae structure became unclear in more than half of its area.

### RNA analysis

Total RNA (1 μg) was isolated from tissue samples with RNA-Bee (TEL-TEST Inc.). Real-time PCR (qPCR) was performed by using a Light Cycler 480 (Roche) with the Universal Probe Library and the Light Cycler 480 Probes Master (Roche) according to the manufacturer’s directions using the following primers:

- *Actb*; 5′-CTAAGGCCAACCGTGAAAAG-3′, 5′-ACCAGAGGCATACAGGGACA-3′.
- *Cidea* 5′-TTCAAGGCCGTGTTAAGGA-3′, 5′-CCTTTGGTGCTAGGCTTGG-3′.
- *Cpt1b* 5′-GAGTGACTGGTGGGAAGAATATG-3′, 5′-GCTGCTTGCACATTTGTGTT-3′.
- *Egln3* 5′-TGTCTGGTACTTCGATGCTGA-3′, 5′-AGCAAGAGCAGATTCAGTTTTTC-3′.
- *Fmo2*; 5′-TTGACGCTGTTATGGTTTGC-3′, 5′-ATACTGGCCTCGGAACCTCT-3′.
- *Mfn1*; 5′-GTGAGCTTCACCAGTGCAAA-3′, 5′-CACAGTCGAGCAAAAGTAGTGG-3′.
- *Mfn2*; 5′-CGAGGCTCTGGATTCACTTC-3′, 5′-CAACCAGCCAGCTTTATTCC-3′.
- *Mtco1*; 5′-CAGACCGCAACCTAAACACA-3′, 5′-TTCTGGGTGCCCAAAGAAT-3′.
- *Mtco2* 5′-AGGCCGACTAAATCAAGCAA-3′, 5′-TCAGAGCATTGGCCATAGAA-3′.
- *Mtco3* 5′-TAGCCTCGTACCAACACATGA-3′, 5′-AGTGGTGAAATTCCTGTTGGA-3′.
- *Mtnd5* 5′-AGCATTCGGAAGCATCTTTG-3′, 5′-TTGTGAGGACTGGAATGCTG-3′.
- *Ppargc1a* 5′-TGAAAGGGCCAAACAGAGAG-3′, 5′-GTAAATCACACGGCGCTCTT-3′.
- *Rplp0* 5′-GATGCCCAGGGAAGACAG-3′, 5′-ACAATGAAGCATTTTGGATAA-3′.
- *Ucp1* 5′-GGCCTCTACGACTCAGTCCA-3′, 5′-TAAGCCGGCTGAGATCTTGT-3′.
- *Actb* or *Rplp0* was used as the internal control.

### RNA sequencing analysis

Total RNA was isolated from tissue samples with an RNeasy Mini Kit (Qiagen, 74104) and its quality was assessed by using the Agilent 2100 Bioanalyzer with the Agilent RNA6000 pico Kit (Agilent Technologies). The TruSeq Stranded mRNA LT Sample Prep Kit (Illumina) was employed to construct four libraries according to the specifications of the manufacturer. Then these libraries were analyzed on a NextSeq500 with a NextSeq 500/550 High Output Kit v2 (Illumina). TopHat 2 (version 2.0.13) was used for mapping reads to the reference genome (Ensembl GRCm38/mm10) with annotation data downloaded from the Ensembl Asia website (URL https://asia.ensembl.org/). Expression of each transcript was quantified as the number of fragments per kilobase of transcript per million fragments mapped (FPKM), and expression was compared among 3 groups by Cuffdiff (included in Cufflinks version 2.2.1).

### Western blot analysis

Whole-cell lysates and mitochondrial lysates were prepared in lysis buffer (10 mM Tris–HCl, pH 8, 140 mM NaCl, 5 mM EDTA, 0.025% NaN_3_, 1% Triton X-100, 1% deoxycholate, 0.1% SDS, and 1 × Complete Protease Inhibitor Cocktail (Santa Cruz, sc-29131)). The lysates (25–50 μg) were resolved by SDS-PAGE and proteins were transferred to PVDF membranes (Millipore). Membranes were incubated with the primary antibody, followed by incubation with horseradish peroxidase-conjugated anti-rabbit immunoglobulin G (Jackson Immunoresearch, #111-035-003), and proteins were detected by enhanced chemiluminescence (GE). The primary antibodies for western blotting were anti-MTCO1 (COX I) antibody (Abcam, ab203912), anti-UCP1 antibody (Abcam, ab10983) and anti-Tomm20 antibody (Abcam, ab186734). These antibodies were used at a dilution of 1:1000.

### Proteomic analysis

The mitochondrial fractions prepared from cardiac tissue of mice treated with TMAO or PBS (control) were subjected to SDS-PAGE, followed by digestion with trypsin before mass spectrometric analysis. Briefly, lanes corresponding to TMAO or the control sample on SDS-PAGE gels (mini gels) stained with Coomassie Brilliant blue were cut into 7 sections of equal size, and in-gel digestion was done with trypsin (Sigma, T6567, Proteomics sequencing grade), essentially according to the method of Katayama et al. (Rapid Commun Mass Spectrom 2001). Peptides from each sample were dissolved in 20 μl of 0.3% formic acid, after which aliquots (2 or 4 μl) were injected into a nano-flow liquid chromatograph (Eksigent nanlLC 415 with ekspert cHiPLC, Sciex) coupled with a tandem mass spectrometer (TripleTOF5600+, Sciex) via a nano-ESI ion source. Duplicate analysis of each sample was conducted in “trap and elute” mode using a ChromeXP C18 Chip column (200 μm × 0.5 mm) as the trap column and a ChromeXP C18 Chip column (75 μm × 150 mm) as the analytical column. Mobile phase A was 0.1% formic acid and mobile phase B was 0.1% formic acid in acetonitrile. Peptides were eluted with a gradient of mobile phase B from 2 to 32% over 40 min at 300 nl/min. Raw data generated by Analyst TF1.6 (Build 6211) were converted to generic Mascot files by MS Converter (Sciex). The two Mascot files generated from duplicate runs of each sample were merged and compared with the mouse protein sequence database in UniProtKB (released May 2015) using the ESI-QUAD-TOF setting. Modifications to the settings were as follows: carbamidomethylation of cysteine was the fixed modification, while the variable modifications were deamidation of asparagine and/or glutamine, N-terminal glutamine to pyroglutamate, N-terminal glutamate to pyroglutamate, oxidation of methionine, phosphorylation of serine and/or threonine, phosphorylation of tyrosine, and methylation of lysine and/or arginine. A maximum of two missed cleavages was allowed. The target false discovery rate was < 1%. Quantitative analysis of COXI protein was performed with Skyline 4.2 software developed by MacCoss Lab at the University of Washington (Seattle, WA) (https://skyline.ms/project/home). Two unambiguously identified peptides (VFSWLATLHGGNIK and EVMSVSYASTNLEWLHGCPPPYHTFEEPTYVK) from COXI protein were used as targets. The Mascot search result files (.dat) were imported to generate spectral libraries. Raw files (.wiff and .wiff.scan) were imported and matched to validated peptides in the spectral libraries, and then were used to generate extracted ion chromatograms (XICs) of the target peptide ions, consisting of precursors and their three isotopic peaks (M, M + 1, M + 2, and M + 3). The total peak area of these precursor peptides was determined.

### Extracellular flux assay

The mitochondrial oxygen consumption rate was measured with a Seahorse XF extracellular flux analyzer, as indicated by the manufacturer (Agilent Technologies) and as reported previously^38,39^. Hearts from mice treated with TMAO (25 mg/kg/day) or PBS were minced on ice in MSHE + BSA (70 mM sucrose, 210 mM mannitol, 5 mM HEPES, 1 mM EGTA, and 0.5% (w/v) fatty acid-free BSA, pH 7.2). Then the tissue was disrupted using 7 strokes with a drill-driven Dounce homogenizer, after which the homogenate was centrifuged at 800×*g* for 10 min at 4 °C. Next, the supernatant was filtered through a 70 μm nylon mesh and centrifuged at 8000×*g* for 10 min at 4 °C. The pellet was resuspended in MSHE + BSA, centrifugation was repeated, and the final pellet was resuspended in a minimal volume of MSHE + BSA. Total protein (mg/ml) was determined using a BCA Protein Assay Kit (Thermo Fisher, 23225). Then 5 μg of the mitochondrial fraction was diluted in 50 μl of 1 × MAS + substrate (70 mM sucrose, 220 mM mannitol, 10 mM KH_2_PO_4_, 5 mM MgCl_2_, 2 mM HEPES, 1.0 mM EGTA, and 0.2% (w/v) fatty acid-free BSA, pH 7.2), and 50 μl of the resulting mitochondrial suspension was added to each well of an XF24 plate. The plate was spun at 2000×*g* for 20 min and 450 μl of 1X MAS + substrate was added to each well. Then the plate was incubated at 37 °C for 8 min for warming and was transferred to the XF24 Analyzer. For the electron flow assay, mitochondria were initially incubated with 10 mM pyruvate (Wako, 199-03062) and 2 mM malate (Wako, 130-00492) as substrates, after which 4 μM FCCP (Santa Cruz, sc-203578), 2 μM rotenone (Sigma, R8875), 10 mM succinate (Wako, 049-28132), 4 μM antimycin A (Sigma, A8674), 10 mM ascorbate (Sigma, A7506), and 100 μM TMPD (N1,N1,N1,N1-tetramethyl-1,4-phenylene diamine; Sigma, 87890) were added in the following steps. The oxygen consumption rate (OCR) was adjusted by mtDNA copy number.

### Enzyme prediction

Candidate enzymes with a role in choline metabolism could not be found by a homology search. Therefore, the E-zyme2^40^ prediction tool, which was developed to predict the enzyme catalyzing an enzymatic reaction from the structures of two chemical compounds, was used to identify candidate enzymes for choline metabolism. E-zyme2 was employed after input of two KEGG compound identifiers, choline (C00114) and tma (C00565), and candidate enzymes were obtained.

### Statistical analysis

Statistical analyses were done with SPSS version 24 software. All values were included in the analyses. If analyses did not reach statistical significance, in some cases outliers (shown as circles in figures) and abnormal values (shown as triangles in figures) were detected by SPSS boxplot analyses (boxplots show the upper whisker, upper quartile, median, lower quartile, and lower whisker) and excluded from further analyses (information described in the Excel raw data files). All outliers and abnormal values were also included in the Excel raw data files. If abnormal values were out of range, they were not shown in the figures and only included in the Excel format raw data files. Non-significant (NS) values in the figures indicate that these analyses included or excluded outlier and/or abnormal values and still did not reach statistical significance. All data are from different biological replicates, and are shown as the mean ± SEM. Differences between groups were examined by the two-tailed Student’s t-test or two-way ANOVA, followed by Tukey’s multiple comparison test, the non-parametric Kruskal Wallis test, or Dunnett’s test for comparison among three or more groups. Survival curves were calculated by the Kaplan–Meier method and were compared with the log-rank test. Data from some experiments were analysed by 2-way repeated measures ANOVA, followed by Tukey’s multiple comparison test. In all analyses, P < 0.05 was considered statistically significant.

## Supplementary Information

Supplementary Figures.

## Data Availability

All data are available from the authors upon reasonable request. Additional material including source data is available online. Gene expression data obtained in these studies were deposited in the Gene Expression Omnibus database (GSE129756). The mass spectrometry proteomics data were deposited in the ProteomeXchange Consortium via jPOST^41^ with the dataset identifier PXD013335.
