# Supplementary material for: Brown adipose tissue dysfunction promotes heart failure via a trimethylamine N-oxide-dependent mechanism
Source: Sci Rep. 2022 Sep 1;12:14883. doi: 10.1038/s41598-022-19245-x (PMC9436957; doi:10.1038/s41598-022-19245-x)
Supplement: Supplementary file 1 — Supplementary Figures. [file 41598_2022_19245_MOESM1_ESM.pdf]

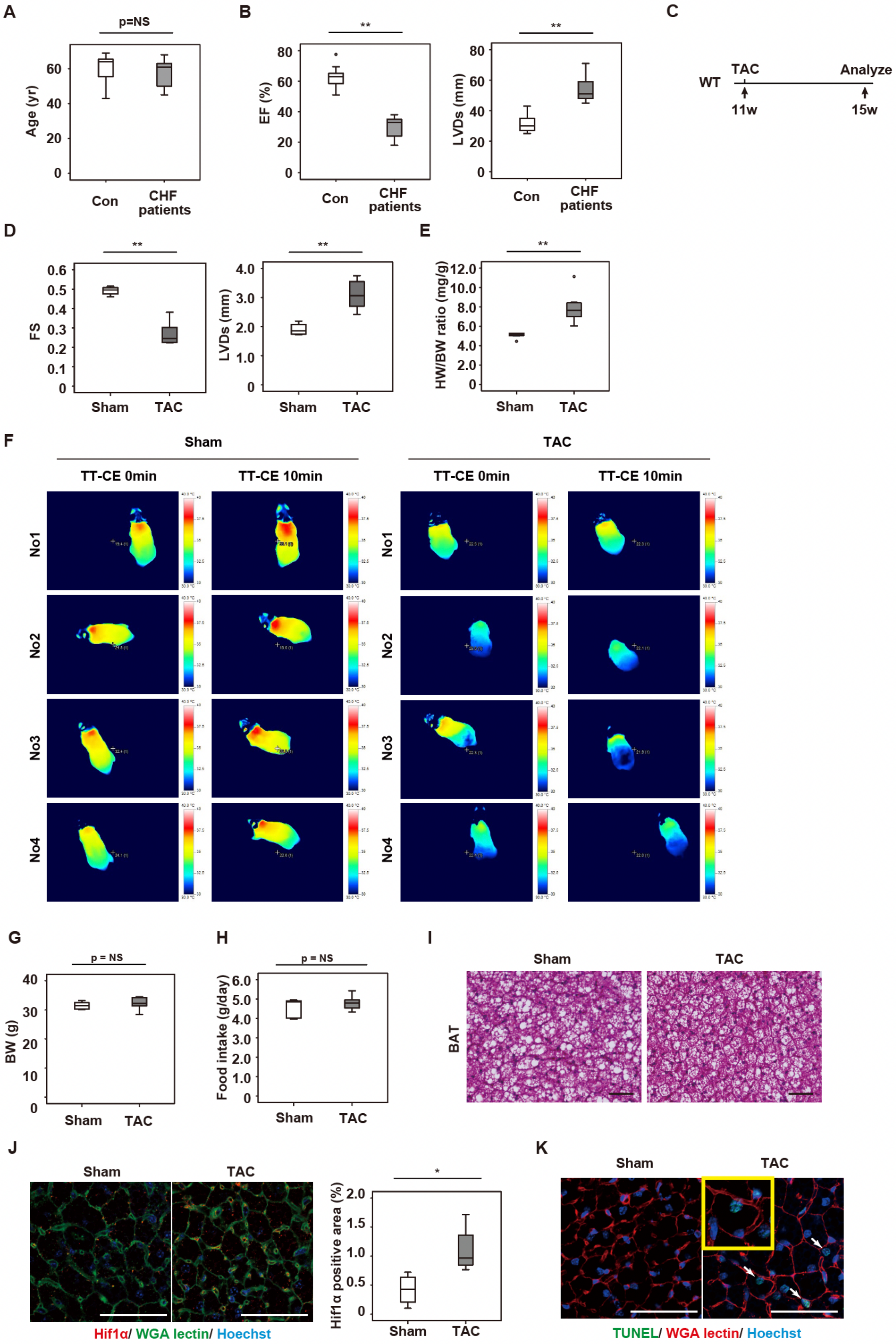

Supplementary Figure 1. Thermogenic response is reduced during LV-pressure overload.

(A) Age of control subjects (Con) or patients with congestive heart failure (CHF) (*n*=15, 9). (B) Cardiac function of control subjects or CHF patients. EF; Ejection fraction (*n* =15, 9), LVDs; left-ventricular systolic dimension (*n* =15, 9). (C) Scheme showing an outline of the experiment. TAC (thoracic aortic constriction) was performed in WT mice at 11 weeks of age. At 4 weeks after TAC, mice were subjected to analyses otherwise mentioned. (D) Cardiac function of mice prepared as described in Figure 1C (FS: fractional shortening; *n* = 4, 6, LVDs: left-ventricular systolic dimension; *n* = 4, 6). (E) Body weight-adjusted heart weight of mice prepared as described in Figure 1C (*n* = 6, 7). (F) Thermographic measurement of body surface temperature after tail tip cold exposure (TT-CE) in TAC model mice. (G, H) Body weight (G) (*n* =6, 7) and food intake (H) (*n* =5, 7) of mice prepared as described in Figure 1C. (I) Hematoxylin and eosin (HE) staining of BAT from mice as prepared in Figure 1C. Scale bar=50 μm. (J) Immunofluorescent staining showing Hif1α-positive (red) cells in BAT after TAC. The graph on the right displays the Hif1α-positive area (%) (*n* = 4, 4). Scale bar = 50 μm. (K) Representative photomicrographs of TUNEL staining in BAT after TAC shown in Figure 1F. Scale bar = 50 μm. Data were analysed by a two-tailed Student's *t*-test (A, B, D, E, G, H and J). *P < 0.05, **P < 0.01. Values are shown as the mean ± s.e.m. NS = not significant.

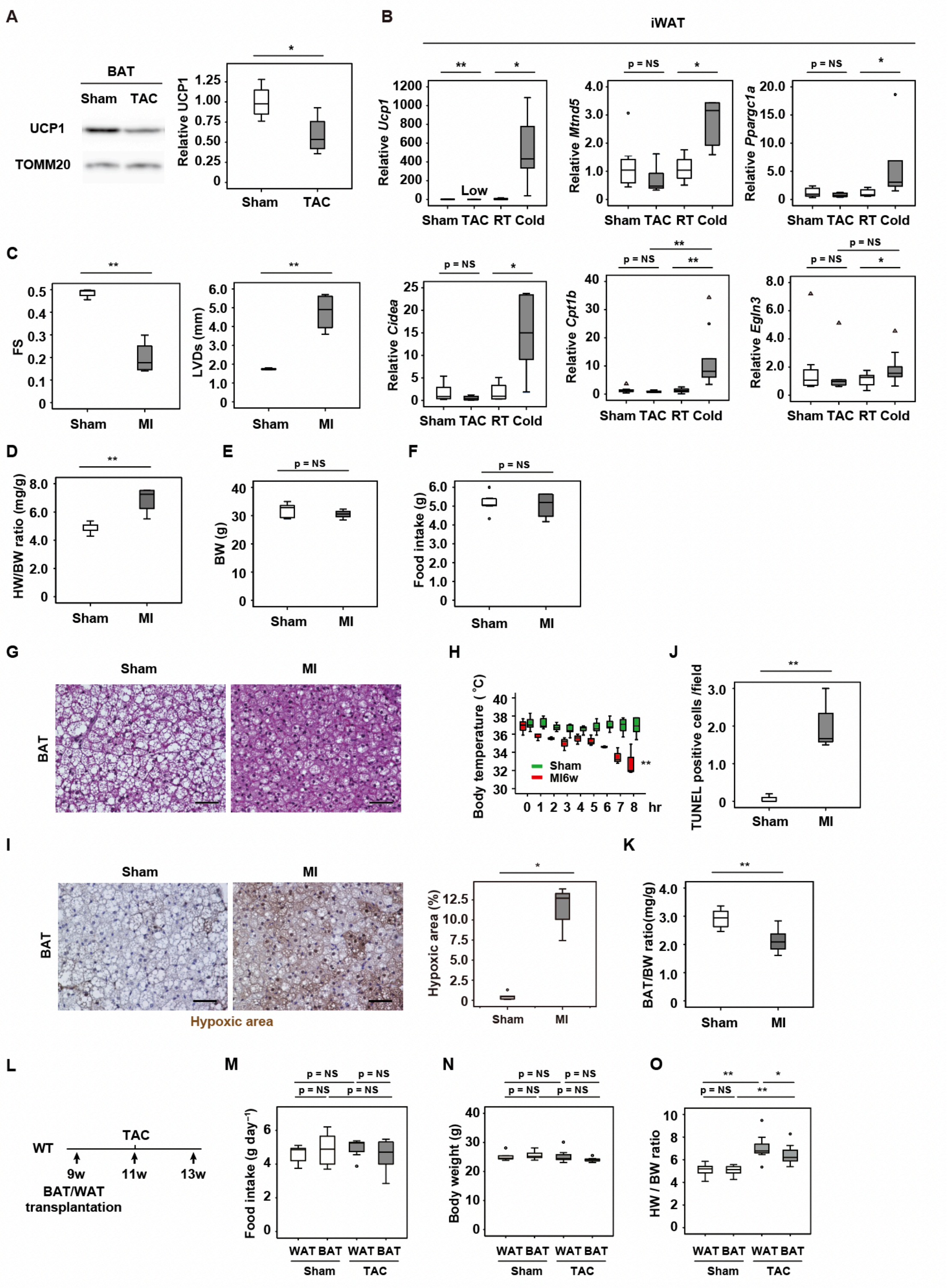

Supplementary Figure 2. Brown adipose tissue thermogenic response is reduced after myocardial infarction.

(A) Western blot analysis of UCP1 in BAT as described in Figure 1C. TOMM20 was used as the loading control. The right panel shows quantification of the data (*n* = 4, 4).

Original blots are presented in Supplementary Figure 10. (B) Relative transcripts assessing beige markers in inguinal WAT (iWAT) of mice 4 weeks after TAC or 1 week after cold exposure (*n*=8,8,6,5 for Ucp1, Mtnd5, Ppargc1a and Cidea, *n*=8,8,11,11 for Cpt1b and Egln3). (C) Cardiac function of mice at 6 weeks after myocardial infarction (MI) (FS; *n*=3, 4, LVDs; *n* =3, 4). (D-F) Body weight-adjusted heart weight (D) (*n* = 5, 4), body weight (E) (*n* = 6, 6), or food intake (F) (*n* = 7, 4) of mice. (G, I) Haematoxylin and eosin (H&E) staining (G) or Pimonidazole staining performed by the Hypoxyprobe-1 method (I) of BAT from mice. The right panel shows quantification of the hypoxic area (*n* = 5, 3). Scale bar = 50 μm. (H) Acute cold tolerance test (*n* = 5, 3). (J) Quantification of TUNEL-positive cells in BAT from mice (*n* = 3, 3). (K) Body weight-adjusted BAT weight of mice (*n* =6, 6). (L) Scheme showing an outline of the experiment. Brown adipose tissue (BAT) or white adipose tissue (WAT) was transplanted into a visceral cavity of WT mice at 9 weeks of age, and TAC was performed at 11 weeks of age. At 2 weeks after TAC, mice were subjected to analyses. (M) Food intake of mice subjected to WAT or BAT implantation as described in Figure 1I (*n* = 5, 7, 7, 10). (N, O) Body weight (N) (*n* = 8, 9, 8, 12) or body weight-adjusted heart weight (O) of mice prepared as described in Figure 1I (*n* = 12, 13, 10, 16). Data were analysed by the 2-tailed Student's t-test (A-F, I, J and K), 2-way ANOVA followed by Tukey's multiple comparison test (M-O), or repeated measures followed by Tukey's multiple comparison test (H). *P<0.05, **P<0.01. Values are shown as the mean ± s.e.m. NS = not significant.

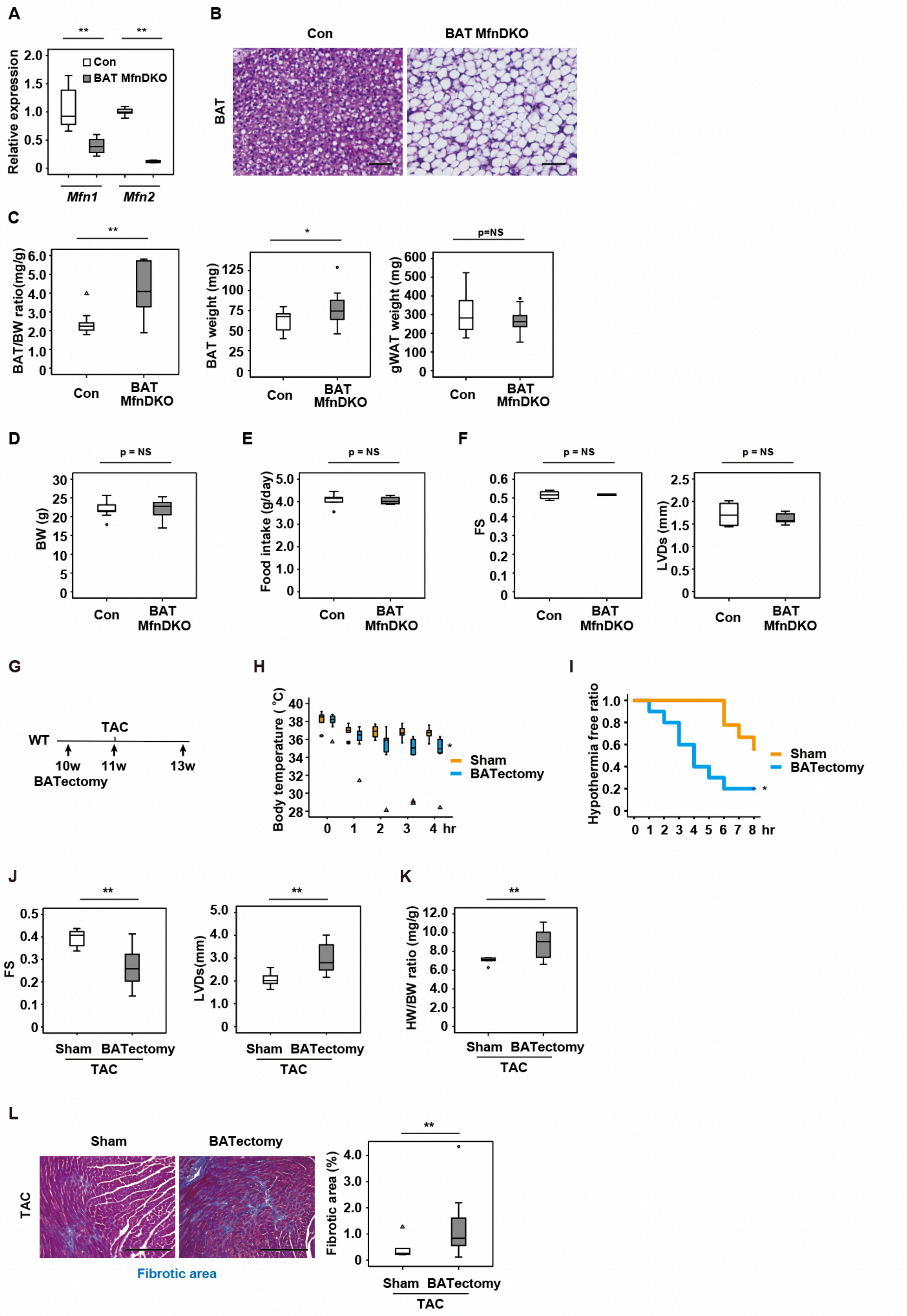

Supplementary Figure 3. Brown adipose tissue dysfunction deteriorates cardiac function after TAC.

(A) Transcripts for mitofusin-1 (*Mfn1*) and mitofusin-2 (*Mfn2*) in BAT from mice prepared as described in Figure 2A (n = 6, 4). (B) Haematoxylin and eosin (H&E) staining (higher magnification) of BAT from mice prepared in Figure 2A. Scale bar = 50 µm. (C, D) Body weight-adjusted BAT weight (C; left) (n=11, 10), BAT weight (C; middle) (n=12, 10), WAT weight (C; right) (n=12, 10) and body weight (D) (n=11, 10) in littermate control mice (Con) and BAT *Mfn*DKO mice aged 8–10 weeks. (E) Food intake of 12-week-old Con or *Mfn*DKO mice (n = 5, 4). (F) Cardiac function of the mice (FS: fractional shortening; n = 4, 5, LVDs: left-ventricular systolic dimension; n = 4, 5). (G) Scheme showing an outline of the experiment. BAT was removed (BATectomy) at 10 weeks of age, and TAC was performed at 11 weeks of age. At 2 weeks after TAC, mice were subjected to analyses otherwise mentioned. (H, I) An acute cold tolerance test (H) (n = 9, 10) and measurement of the hypothermia-free ratio in this study (I) (n = 9, 10) were performed 1 week after BATectomy (this experiment was done before TAC). Sham indicates sham operation for BATectomy. (J) Assessment of cardiac function in the mice at 2 weeks after TAC (n = 10, 14). (K) Body weight-adjusted heart weight of mice (n = 6, 13). (L) Masson's trichrome staining of hearts. The right panel shows quantification of the fibrotic areas (n = 6, 13). Scale bar = 50 µm. Data were analysed by the 2-tailed Student's t-test (A, C-F and J-L), repeated measures followed by Tukey's multiple comparison test (H), or the log-rank test for Kaplan-Meier method (I). *P < 0.05, **P < 0.01. Values are shown as the mean ± s.e.m. NS = not significant.

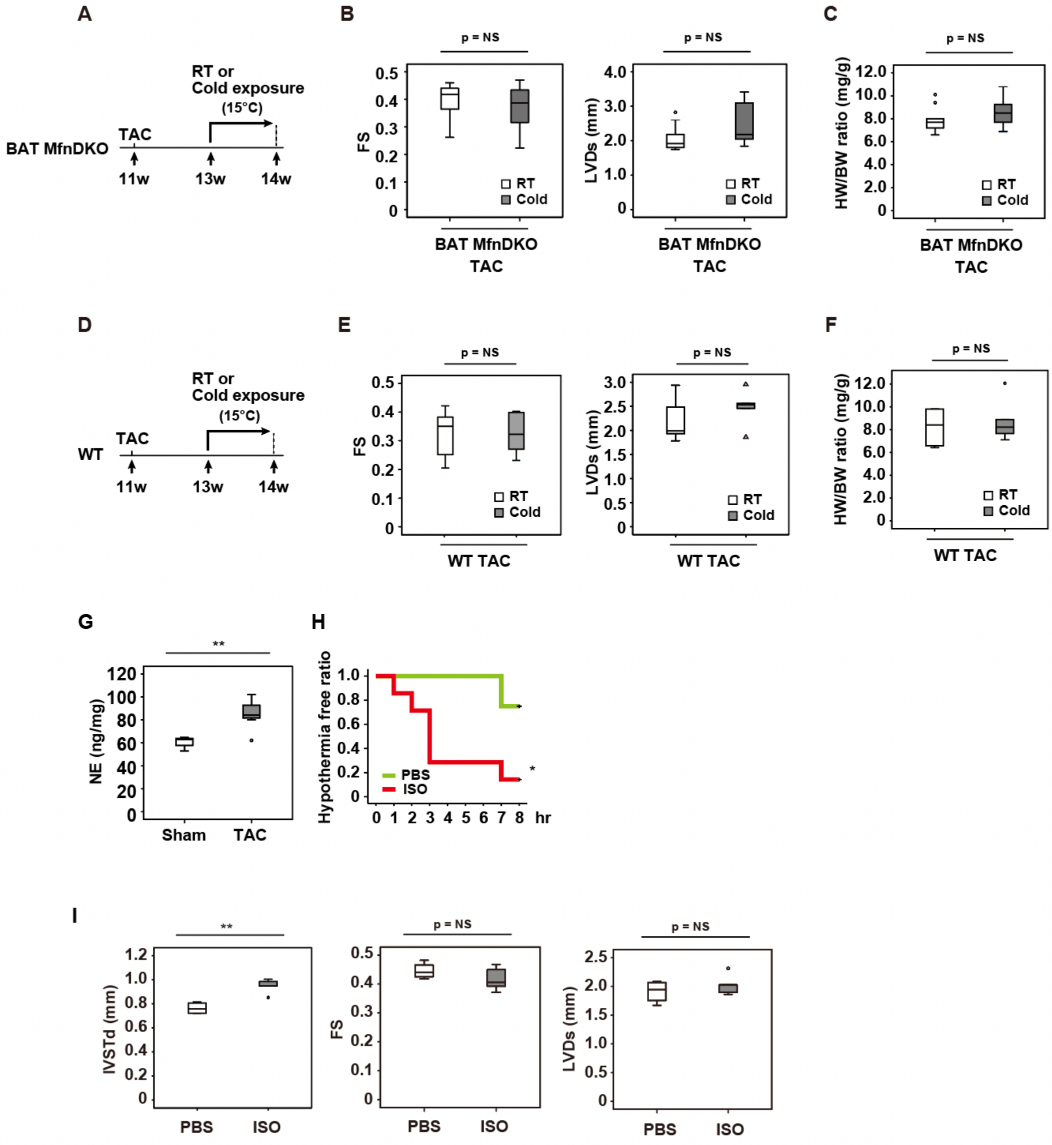

Supplementary Figure 4. Modulation of environmental temperature *per se* has little influence on heart failure.

(A) Scheme showing an outline of the experiment. At 2 weeks after TAC, BAT MfnDKO mice were subjected to chronic cold exposure (15°C) (Cold) or room temperature (RT) for 1 week. (B) Cardiac function of mice prepared as described in Figure S4A (FS: fractional shortening, LVDs: left-ventricular systolic dimension) (*n*=9, 7). (C) Body weight-adjusted heart weight of the mice (*n* =9, 7). (D) Scheme showing an outline of the experiment. At 2 weeks after TAC, wild-type (WT) mice were subjected to chronic cold exposure (15°C) (Cold) or room temperature (RT) for 1 week. (E) Cardiac function of mice prepared as described in Figure S4D (*n* =7, 6). (F) Body weight-adjusted heart weight of the mice (*n* =7, 6). (G) Norepinephrine (NE) level in BAT from WT mice at 4 weeks after Sham or TAC (*n*=4, 7). (H) Hypothermia-free ratio in the acute cold tolerance

test in WT mice treated with saline (PBS) or isoproterenol (ISO) (n=4, 7). (I) Cardiac function of mice prepared as described in Fig. S4H (n=4, 5). IVSTd; interventricular septal thickness, diastolic, FS; fractional shortening, LVDs; left-ventricular systolic dimension. Data were analysed by the 2-tailed Student's t-test (B, C, E-G and I) or the log-rank test for Kaplan-Meier method (H). *$P < 0.05$, **$P < 0.01$. Values are shown as the mean ± s.e.m. NS = not significant.

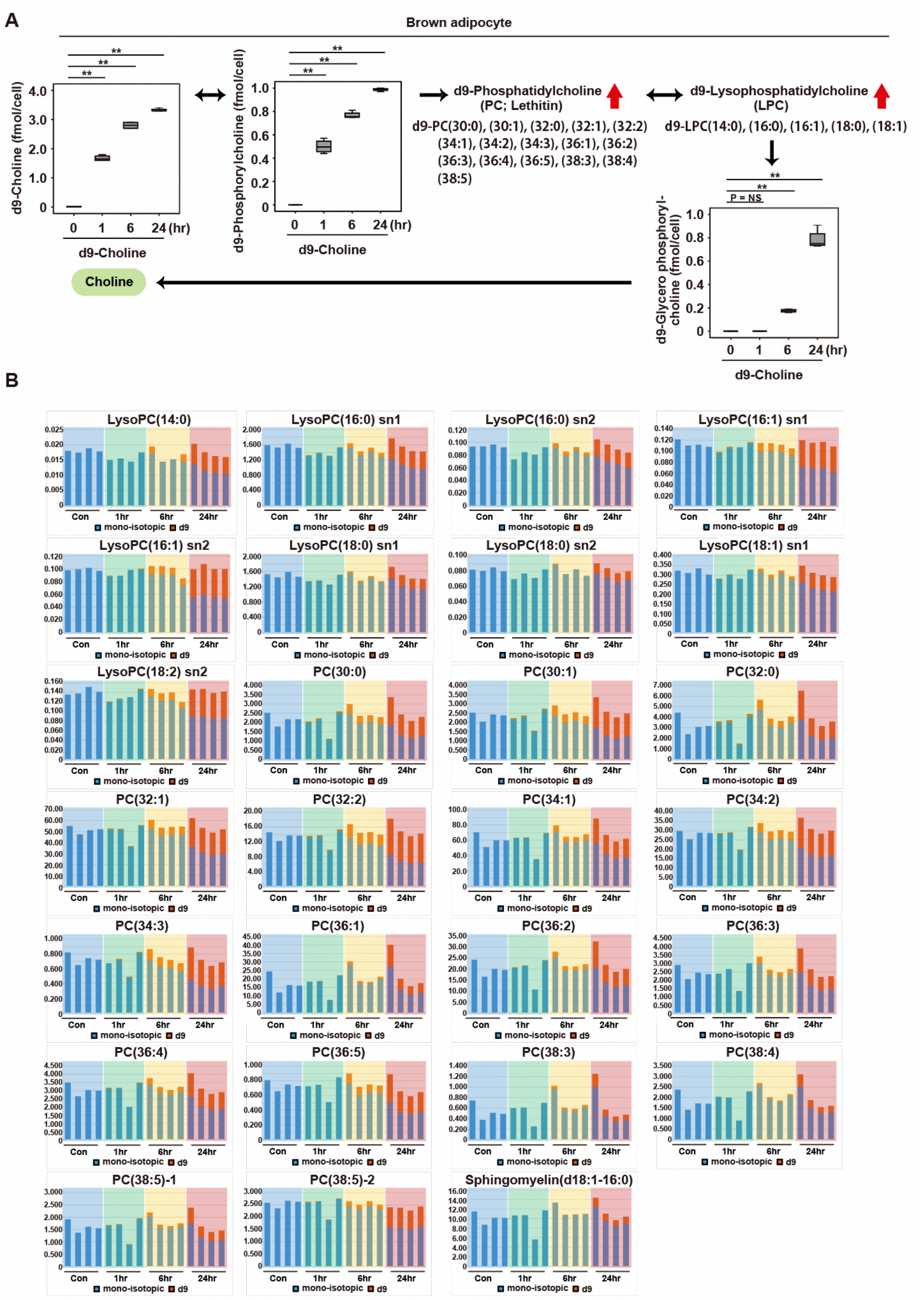

Supplementary Figure 5. Metabolomic flux studies in differentiated brown adipocytes.

(A) Metabolomic flux analysis of differentiated brown adipocytes treated with d9-choline (*n*=4, 4, 4, 4). Data for d9-choline, d9-phosphorylcholine and d9-glycerophosphorylcholine are described in a box plot. Right panels show the summary of d9-phosphatidylcholines (PC) and d9-lysophosphatidylcholines (LPC) showing an increase with d9-choline. (B) Lipidomic flux analysis was performed in differentiated brown adipocytes using d9-choline at the indicated times. Bars in the graph show individual samples. A summary is shown in Figure S5A. Data were analysed by 2-way ANOVA followed by Dunnett's comparison test (A). *P<0.05, **P<0.01. Values are shown as the mean ± s.e.m. NS = not significant.

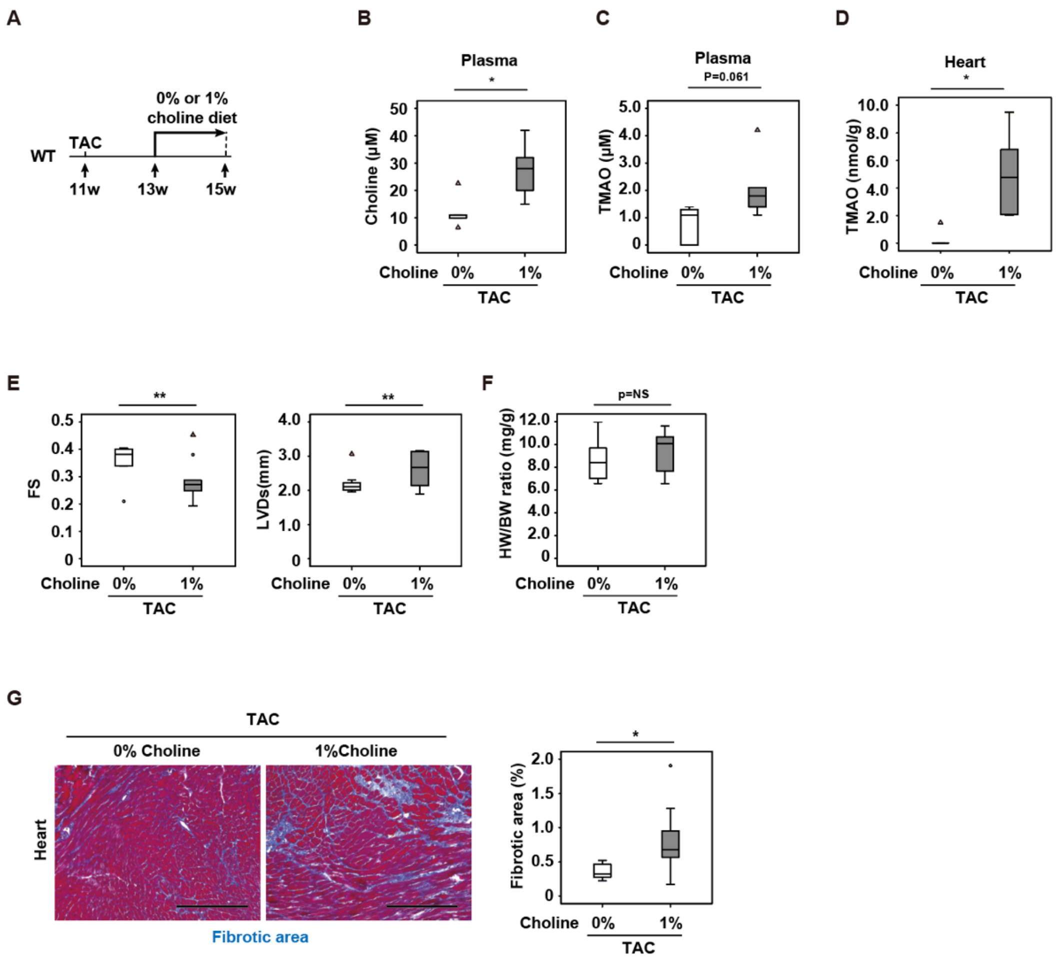

Supplementary Figure 6. Dietary choline administration deteriorates cardiac dysfunction after TAC.

(A) Scheme showing an outline of the experiment. At 2 weeks after TAC, WT mice were subjected to administration of a high- (1%) or low-choline (0%) diet for 2 weeks. (B-D) Plasma choline (B) and TMAO (C), heart TMAO (D) levels in mice (n = 5, 5). (E) Cardiac function as shown by fractional shortening (FS) and left-ventricular systolic dimension (LVDS) (n = 8, 10). (F) Body weight-adjusted heart weight (n = 6, 10). (G) Masson's trichrome staining of myocardium. The right panel shows quantification of the fibrotic area (n = 6, 10). Scale bar = 50 μm. Data were analysed by a 2-tailed Student's t-test (B-G). *$P < 0.05$, **$P < 0.01$. Values are shown as the mean ± s.e.m. NS = not significant.

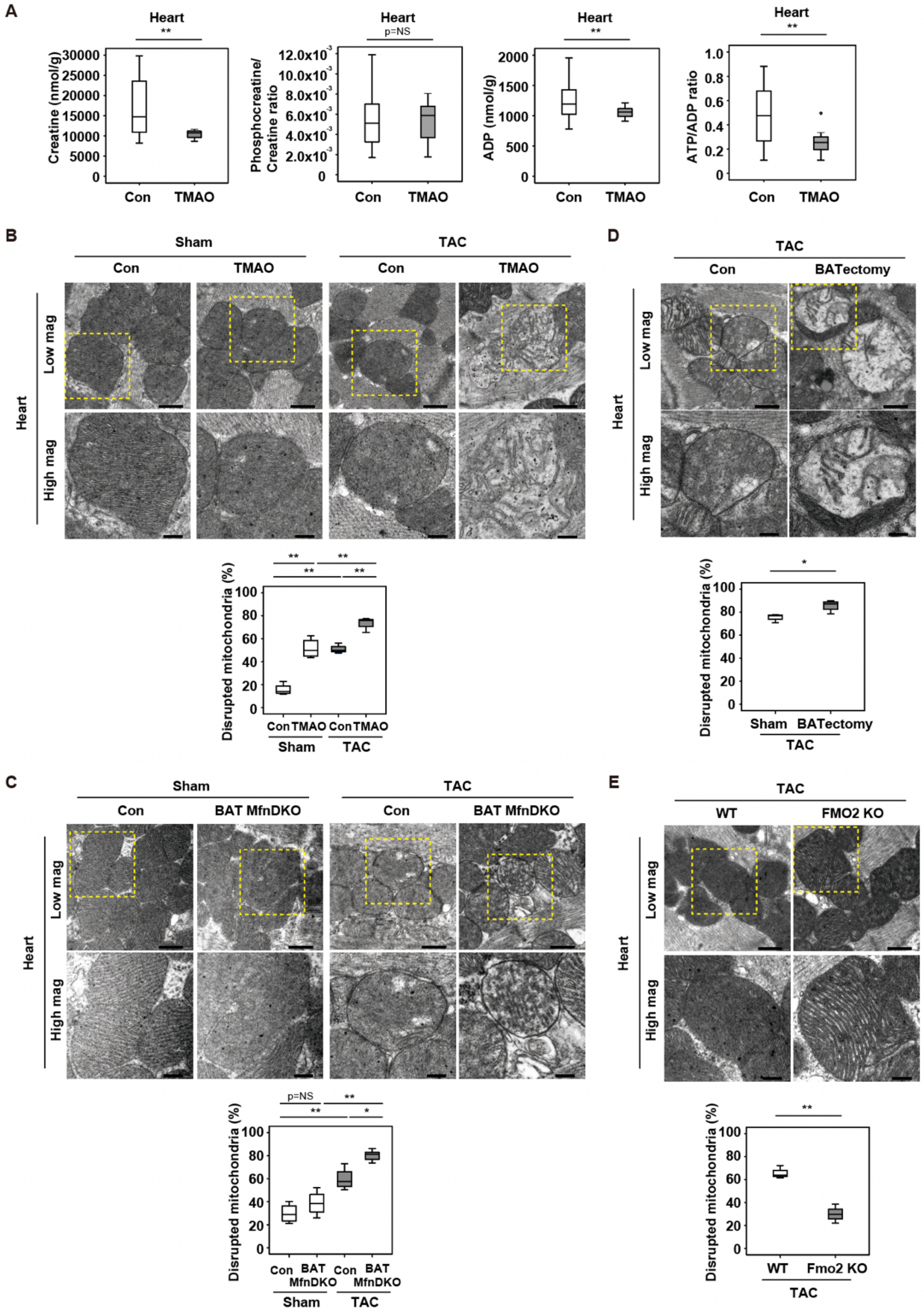

Supplementary Figure 7. Trimethylamine N-oxide induces mitochondrial dysfunction in the heart.

(A) Metabolomic analysis of the myocardium of mice administered PBS (Con) or trimethylamine N-oxide (TMAO) for 2 weeks (n = 24, 11). (B-E) Transmission electron

microscopy of cardiac tissues from wild-type mice subjected to sham surgery or TAC with or without TMAO introduction (B), littermate control (Con) mice or BAT Mfn DKO mice 4 weeks after sham or TAC surgery (C), BATectomy mice subjected to TAC (D), or systemic Fmo2 knockout mice subjected to TAC (E). The lower panels show quantification of the disrupted mitochondria (B: n = 5, 5, 3, 3, C: n = 4, 4, 4, 5, D: n = 3, 4, E: n = 3, 3). Scale bar=500 nm for low magnification and 200 nm for high magnification. Data were analysed by the 2-tailed Student's t-test (A, D and E) or 2-way ANOVA followed by Tukey's multiple comparison test (B and C). *$P < 0.05$, **$P < 0.01$. Values are shown as the mean ± s.e.m. NS = not significant.

A

GO terms with negative enrichment in TMAO compared to con (x0.5)

pathway ID	pathway description	gene count	false discovery rate
GO.0009987	cellular process	72	0.0349
GO.0065007	biological regulation	67	0.00124
GO.0050789	regulation of biological process	65	0.00128
GO.0044763	single-organism cellular process	65	0.00929
GO.0050794	regulation of cellular process	63	0.00123
GO.0008152	**metabolic process**	58	0.0258
GO.0019222	**regulation of metabolic process**	51	0.000815
GO.0048518	positive regulation of biological process	51	5.19E-05
GO.0032501	multicellular organismal process	48	0.00117
GO.0044707	single-multicellular organism process	47	0.00117
GO.0031323	**regulation of cellular metabolic process**	45	0.00161
GO.0050896	response to stimulus	45	0.015
GO.0048856	anatomical structure development	44	0.000353
GO.0060255	**regulation of macromolecule metabolic process**	44	0.00161
GO.0080090	**regulation of primary metabolic process**	44	0.00165

(Totally 400 genes and 175 GO terms)

GO terms with positive enrichment in TMAO compared to con (x2)

pathway ID	pathway description	gene count	false discovery rate
GO.0044699	single-organism process	95	0.0285
GO.0044763	single-organism cellular process	87	0.047
GO.0008152	**metabolic process**	82	0.0238
GO.0050789	regulation of biological process	82	0.038
GO.0050794	regulation of cellular process	80	0.0238
GO.0050896	responce to stimulus	79	5.29E-07
GO.0019222	**regulation of metabolic process**	71	0.00012
GO.0032501	multicellular prganismal process	68	0.000113
GO.0048518	positive regulation of biological process	67	1.96E-05
GO.0044707	single-multicellular organism process	67	0.000107
GO.0051716	cellular response to stimulus	66	8.71E-06
GO.0031323	**regulation of cellular metabolic process**	64	0.000234
GO.0080090	**regulation of primary metabolic process**	62	0.000375
GO.0060255	**regulation of macromolecule metabolic process**	61	0.000625
GO.0048522	positive regulation of cellular process	58	0.000156

(Totally 622 genes and 187 GO terms)

B

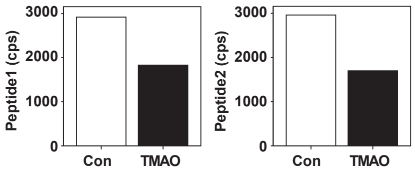

C

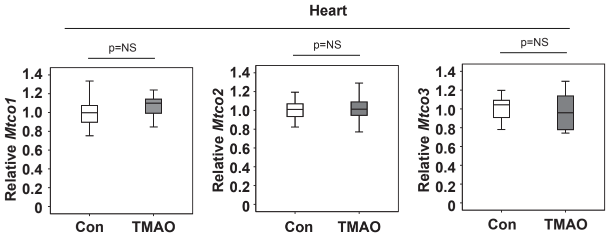

Supplementary Figure 8. COX1 protein is reduced in the heart after TMAO treatment.

(A) Gene ontology (GO) terms with negative (left) and positive (right) enrichment in cardiac tissues from TMAO-treated WT mice compared to control mice. (B) Quantitative data from proteomic analysis for COXI as analysed in Figure 5E (*n*=1,1). Peptide 1 sequence: VFSWLATLHGGNIK, Peptide 2 sequence: EVMSVSYASTNLEWLHGCPPPYHTFEEPTYVK. (C) Transcripts for cytochrome oxidase 1, 2 and 3 (COX1, COX2 and COX3) (*Mtco1*, *Mtco2* and *Mtco3*) in the hearts of WT mice administered PBS (Con) or TMAO for 2 weeks (*n* = 11,11). Data were analysed by the 2-tailed Student's t-test (C). NS = not significant.

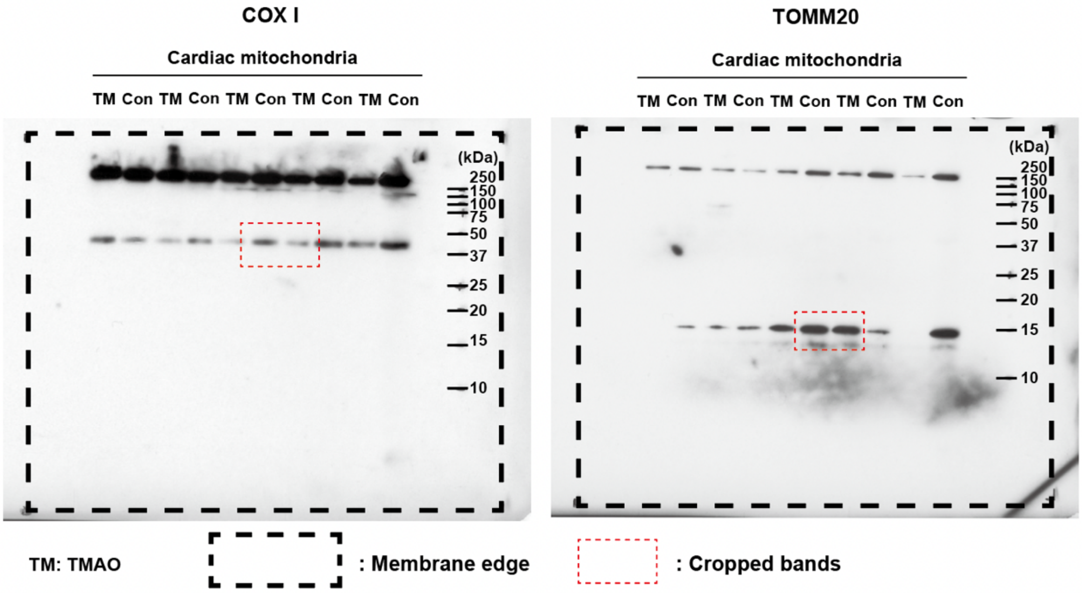

Supplementary Figure 9. Full blots of Figure 5F

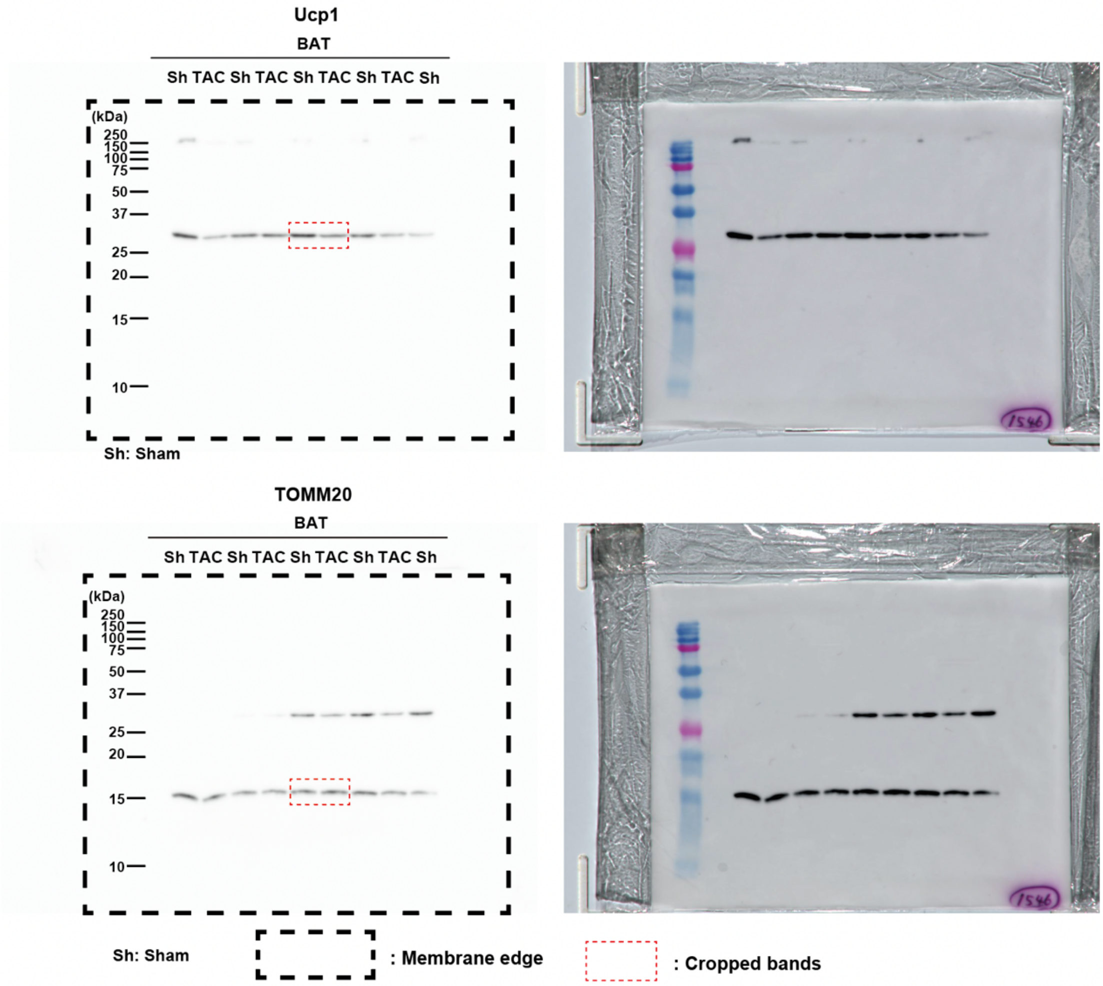

Supplementary Figure 10. Full blot of Supplementary Figure 2F
